# *C. perfringens* enterotoxin-claudin pore complex: Models for structure, mechanism of pore assembly and cation permeability

**DOI:** 10.1016/j.csbj.2024.11.048

**Published:** 2024-12-02

**Authors:** Santhosh Kumar Nagarajan, Joy Weber, Daniel Roderer, Jörg Piontek

**Affiliations:** a Clinical Physiology/Nutritional Medicine, Department of Gastroenterology, Rheumatology and Infectious Diseases, Charité – Universitätsmedizin Berlin, Corporate Member of Freie Universität Berlin and Humboldt-Universität zu Berlin, Hindenburgdamm 30, 12203 Berlin, Germany; b Leibniz-Forschungsinstitut für Molekulare Pharmakologie (FMP), Robert-Rössle-Straße 10, 13125 Berlin, Germany

**Keywords:** *Clostridium perfringens* enterotoxin, Claudin, Pore-forming toxins, Molecular dynamics simulations, Protein complex modeling

## Abstract

The pore-forming *Clostridium perfringens* enterotoxin (CPE), a common cause of foodborne diseases, facilitates Ca^2+^ influx in enterocytes, leading to cell damage. Upon binding to certain claudins (e.g., claudin-4), CPE forms oligomeric pores in the cell membrane. While the mechanism of CPE-claudin interaction is well understood, the structure and assembly of the pore complex remain elusive. Here, we used AlphaFold2 complex prediction, structure alignment, and molecular dynamics simulations to generate models of prepore and pore states of the CPE/claudin-4 complex. We sequentially addressed CPE-claudin, CPE-CPE, and claudin-claudin interactions, along with CPE conformational changes. The CPE pore is a hexameric variant of the typical heptameric pore stem and cap architecture of aerolysin-like β-barrel pore-forming toxins (β-PFT). The pore is lined with three hexa-glutamate rings, which differ from other β-PFTs and confer CPE-specific cation selectivity. Additionally, the pore center is indicated to be anchored by a dodecameric claudin ring formed by a cis-interaction variant of an interface found in claudin-based tight junction strands. Mutation of an interface residue inhibited CPE-mediated cell damage in vitro. We propose that this claudin ring constitutes an anchor for a twisting mechanism that drives extension and membrane insertion of the CPE β-hairpins. Our pore model agrees with previous key experimental data and provides insights into the structural mechanisms of CPE-mediated cytotoxic cation influx.

## Highlights

• *C. perfringens* enterotoxin binds claudins and forms cytotoxic pores in cell membranes.
• Structural models of the toxin-claudin pore complex were generated and simulated.
• Three hexa-glutamate rings of the hexameric β-barrel pore confer cation selectivity.
• A potential mechanism for prepore formation and prepore-pore transition is provided.
• A 12-claudin ring is proposed to anchor the toxin and drive pore formation.

## Graphical Abstract

**Figure:**
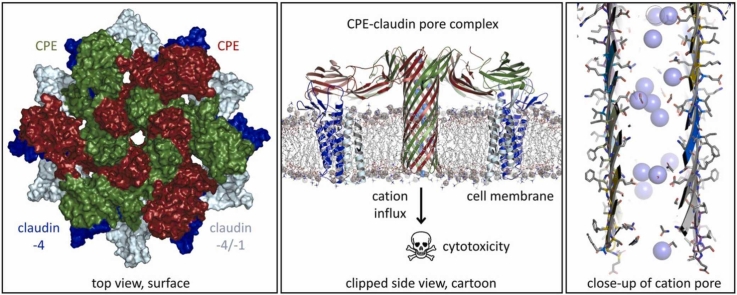

## 1 Introduction

*Clostridium perfringens*, an anaerobic, Gram-positive, spore-forming bacterium, is known to cause a variety of illnesses worldwide, such as gas gangrene, wound infections, and various diseases affecting the gastrointestinal system of both humans and animals [1]. Its toxicity derives from a series of at least 16 toxins produced by different strains [2].

Infection with *C. perfringens* type F (formerly type A) is responsible for one of the most common foodborne diseases worldwide, caused by release of the *C. perfringens* enterotoxin (CPE) [3], [4]. CPE has been shown to cause characteristic gastrointestinal symptoms [5]. These are typically short-term but can result in serious complications, such as lethal necrotizing colitis [6]. In addition, CPE has been linked to non-foodborne diseases such as antibiotic-associated diarrhea [7], similar to toxins of *Clostridioides difficile* [8].

After bacterial ingestion, usually through the consumption of contaminated food, the sporulation process takes place in the intestine. Thereby, the enterotoxin is strongly expressed and released into the intestinal lumen upon lysis of the bacterial mother cell [2], [9]. CPE binds to intestinal enterocytes through receptors: specific claudins (CLDN), such as CLDN3 and CLDN4, which are abundantly localized at the villus tips of the small intestine [10], [11]. Claudins, a ≥ 27-member family of ∼ 23–34 kDa proteins with four transmembrane (TM) helices and two extracellular segments (ECS), form the backbone of tight junctions, and regulate the paracellular permeability of solutes and water in epithelia and endothelia [12], [13], [14]. Non-junctional claudins can serve as CPE receptors [15], [16], [17]. Within a short time after CPE binds to receptors, severe damage to epithelial cells occurs due to the formation of cytotoxic pores in the plasma membrane [18], [19], [20], [21]. Ca^2+^ influx triggers cell death by necroptosis or apoptosis [22], [23], [24], [25].

CPE functions as a pore-forming toxin (PFT), which relies on oligomerization on cell membranes and insertion of a membrane-spanning segment. CPE has been suggested to belong to the subgroup of β-PFTs that form a β-barrel pore [26]. β-PFTs include *S. aureus* α-toxin family, MACPF/CDC, ABC toxins, aegerolysins, colicins, AB toxins, and the emerging superfamily of aerolysin/ETX-MTX-2 proteins [27], [28]. The N-terminal domain of monomeric CPE (residues 1–205) shows structural homology with the C-terminal pore-forming domain of aerolysin; thus, CPE was assigned to the aerolysin/ETX-MTX-2 superfamily [29].

The soluble CPE monomer (35 kDa) is composed of two main functional domains: (1) the N-terminal domain (nCPE, residues 1–202), which facilitates the toxin’s cytotoxicity by pore formation, and (2) the C-terminal domain (cCPE, residues 203–319), which binds to the claudin receptor on target cells. Crystal structures revealed that cCPE consists of one structural domain (I), while nCPE is made up of two structural domains (II and III). Each of these domains consists of one α-helix and several, mostly antiparallel β-strands (I:9, II:5, III:5 β-strands; Figure S1A) [29], [30].

Biochemical and structural data revealed that the residues Y306, Y310, Y312, and L315, located at the base of a binding pocket of cCPE, are responsible for its nanomolar affinity to certain claudins, namely CLDN3, −4, −6, −7, −8, −9, −14, and −19 [31], [32], [33]. The claudin’s ECS, which form an antiparallel β-sheet composed of five β-strands, constitute the binding site for CPE. The ECS2 portion, consisting of strand β5, and an elongation of TM helix 3, has been shown to bind in the described pocket in cCPE [34], [35], [36], [37]. The strong interaction with CPE is mediated by the CLDN-ECS2 motif (N/D(P_-1_) P/S(P) L/M/V/I/S(P_+1_) V/T(P_+2_) P/A/N/D(P_+3_)); where letters indicate amino acids at positions (P_-1_) to (P_+3_), and P refers to a conserved proline in ECS2, e.g., P150 in CLDN4 (Figure S1D). The more stringent motif (D/E(P_-4_) x(P_-3_) x(P_-2_) N(P_-1_)P(P) L/M/V(P_+1_) V(P_+2_) P/A(P_+3_) was identified for high-affinity interaction [15], [38], [39], [40]. Moreover, the β1-β2 loop of the ECS1 portion contributes to the binding [35], [36], [37].

Upon binding, the cCPE structure remains unchanged. However, according to biochemical data, a non-cytotoxic "small complex" with a molecular weight of ∼ 90 kDa is formed, consisting of CPE and two claudins, at least one of which acts as a CPE receptor [41]. It was further suggested that several of these small complexes oligomerize on the cell surface into a prepore prior to pore formation [42]. Homology of the CPE monomer to the HA3 subcomponent of *C. botulinum* type C progenitor toxin as well as studies of the stoichiometry and mass of the developing prepore or pore complex lead to the hypothesis that six small complexes are involved [29], [41]. Therefore, the resulting 425–500 kDa complex is also referred to as "CPE hexamer-1" (CH-1). In mammals, a heavier, 550–660 kDa CH-2 complex has also been found, which includes occludin, another TJ protein [41]. In contrast to cCPE, the two structural domains of nCPE are proposed to undergo drastic conformational changes upon formation of the cytotoxic pore [29]. An amphipathic region (residues 81–106) in domain II has been suggested as the membrane-spanning region [42]. However, this has not yet been proven by a 3D structure of the pore complex. Due to the proposed conformational change in the N-terminal domain, existing structures of the CPE monomer [29], [30] or of complexes of cCPE or CPE with claudins [36], [43], [44] provide insufficient information on the formation and structure of the final pore.

Here, in line with the existing biochemical data and based on AlphaFold2 and molecular dynamics (MD) simulations, we propose structural models for the CPE prepore as well as the final pore complex.

## 2 Materials and methods

### 2.1 Modeling and MD simulation of CPE/CLDN4 complexes

The Schrödinger Maestro BioLuminate software (BioLuminate, version 4.9.132, Release 2022–4, Schrödinger, LLC, Germany, 2022) was used to model, refine, and simulate the CPE pore–CLDN4 models. All the simulations were conducted on a Linux-x86_64-based GPU workstation. Both BioLuminate and Schrödinger PyMOL 2.5.7 (http://www.pymol.org/) were utilized to create the model representations presented in the figures.

### 2.2 AlphaFold2-based structural prediction of CPE oligomers

For the generation of initial models of the oligomeric CPE pore, the AlphaFold2 and MMseqs2-based prediction platform ColabFold v1.5.5 (https://colab.research.google.com/github/sokrypton/ColabFold/blob/main/AlphaFold2.ipynb) was used with GPUs, extended RAM, and Python 3 [45]. The following input parameters were varied: (a) Sequence: 1–319, 1–202, 26–202, 26–319; (b) number of subunits: 6 or 7; (c) template mode: none, pdb 100, custom; (d) model types: Alphafold2_multimer_v3, Alphafold_ptm, deepfold_v1; (e) pairing strategy: greedy, complete; (f) for other parameters, the default settings were used. The following AlphaFold (AF) prediction runs were used for further analysis (Table 1). Notably, for the state VII-nCPE_6_, two independent prediction runs (AF-IV and AF-V) resulted in a similar structural prediction. Hence, only AF-IV was selected for further analysis.

**Table 1:** AlphaFold (AF) prediction runs.

Prediction run	State name	CPE Sequence (residue numbers)	Sub- units	Template mode	Model types	Pairing strategy	Model rank
AF-I	IV-nCPE_6_	1 −202	6	pdb100	Multimer_v3	greedy	3
AF-II	V-nCPE_6_	1 −202	6	pdb100	Multimer_v3	greedy	4
AF-III	VI-nCPE_6_	26 −319	6	pdb100	Multimer_v3	greedy	3
AF-IV	VII-nCPE_6_	1 −202	6	pdb100	Multimer_v3	greedy	1
AF-V	VII-nCPE_6_	26 −202	6	pdb100	Multimer_v3	greedy	2
AF-VI	VII-nCPE_7_	26 −202	7	pdb100	Multimer_v3	greedy	2

### 2.3 Further modeling, multi subunit assembly and MD simulation of CPE and CPE/CLDN4 complexes

From the structure AF-IV (= state VII-nCPE_6_), the disordered N-terminal region (M1–K25) was removed, the structure was prepared and embedded in a membrane lipid bilayer made up of 1-palmitoyl-2-oleoyl-sn-glycero-3-phosphocholine (POPC) lipids. The positioning of the CPE pores within the membrane relative to the hydrocarbon layer of the lipid bilayer was calculated using the PPM 3.0 web server [46] (https://opm.phar.umich.edu/ppm_server). The membrane-protein complex was placed in an orthorhombic simulation box with buffer distances of 10 Å, 10 Å, and 15 Å in the x-, y-, and z-axes, respectively. TIP3P water molecules [47], charge neutralizing Na^+^ ions, and 0.15 M NaCl were added to generate the simulation system. The simulations were carried out using the Desmond Molecular Dynamics module (Desmond Molecular Dynamics System, D. E. Shaw Research, New York, NY, 2022; Maestro-Desmond Interoperability Tools, Schrödinger, New York, NY, 2022; [48]) within Maestro BioLuminate.

After minimizing the system to a local energy minimum within 200 ps, it was relaxed in an NPγT ensemble to room temperature, pressure, and surface tension – 310 K, 1.01325 bar, 4000 bar × Å. The relaxed system was then equilibrated by stepwise releasing constraints on the protein over ∼ 45 ns. A final production run, free of constraints, was performed for 100 ns. The simulations were performed using an NPT ensemble in OPLS4 force field [49], with the Martyna-Tobias-Klein method [50] and Nosé–Hoover chain method [51] serving as the barostat and thermostat, respectively.

### 2.4 Modeling and MD simulations of CPE pore-CLDN4 complexes

#### 2.4.1 CPE pore complexes containing six CLDN4 subunits

We developed a CPE pore-CLDN4 complex with six claudin subunits, each bound to the cCPE domain of one of the six CPE subunits. Two variants of CPE pore-CLDN4 complex were modeled, differing mainly in the region between residues V187–A204 that link nCPE to cCPE. In one variant (VIIIa-CPE_6_R_6_), this region forms a β-strand, while in the other (VIIIb-CPE_6_R_6_), it forms an α-helical conformation.

VIIIb-CPE_6_R_6_ was based on the AlphaFold prediction AF-III for CPE_26–319_. However, the β-barrel pore in AF-III was not valid, as it would not span the membrane. Hence, from AF-III, only the residues T26–L63 and Y127–F319 from domain II and III (Figure S1), and cCPE (domain I) were used for modeling the CPE/claudin complex. The β-barrel pore structure (N64–E126) from the pore structure simulation (state VII-nCPE_6_ after equilibration) was grafted onto AF-III. The crystal structure of human CLDN4 in complex with cCPE (PDB ID: 7KP4) was then aligned with and replaced the cCPE in each CPE subunit of AF-III-VII-nCPE_6_ fusion. This modeling resulted in a discontinuous ring of six claudins (receptors) anchoring the full-length CPE hexameric pore (complex VIIIb-CPE_6_R_6_).

Similarly, another CPE pore-CLDN4 complex (VIIIa-CPE_6_R_6_) was modeled using the CPE structure AF-IV (= VII-nCPE_6_) instead of AF-III. AF-IV was aligned with VIIIb-CPE_6_R_6_ for identifying the position and subsequent alignment of CLDN4 crystal structures bound with cCPE. One critical difference between VIIIb-CPE_6_R_6_ and VIIIa-CPE_6_R_6_ is that, in VIIIa-CPE_6_R_6_, the residues (K197–A205) that connect cCPE (from VIIIb-CPE_6_R_6_) to nCPE (from AF-IV) were manually modified to facilitate the connection.

Each of the two variants (VIIIa-CPE_6_R_6_ and VIIIb-CPE_6_R_6_) were embedded in a membrane lipid bilayer as described for state nCPE-IV (3.1.). Importantly, the claudin transmembrane helices as well as the CPE pore tips spanned the membrane in both variants. Following a similar MD simulations protocol as for the VII-nCPE_6_, the two complex systems were minimized, relaxed, equilibrated, and then simulated without constraints for 100 ns.

#### 2.4.2 CPE pore complexes containing a 12-claudin ring

A second CPE pore-CLDN4 complex was developed with a ring of 12 CLDN4 subunits to achieve a continuous and closed claudin ring. However, while modeling such a 12-claudin ring based on the 6-claudin ring (VIIIa-CPE_6_R_6_), we noticed that the gap between claudins was so wide that contact between neighboring claudins was not possible. Hence, we modeled a 12-claudin ring with a smaller diameter than that of the 6-claudin ring to form a ring with contacts between claudins that represent a modification of the linear cis-interfaces found in claudin strands. For this, our previously published claudin strand model [52], [53] was used as a template. A dimer from the template was extracted, and two human CLDN4 structures were aligned with the dimer. The missing N-terminal residues (M1–M4) in the structure from PDB ID 7KP4 were grafted from the PDB ID 5B2G. At first, one of the two subunits in the dimer was rotated by ∼30°. This rotation was necessary to generate a 12-claudin ring by replication. More precisely, the rotated dimer was duplicated and subunit 1 from the duplicated dimer was aligned with subunit 2 from the original dimer. Similarly, such rotation and alignment were repeated six times. This resulted in a 12-claudin ring connected through an alternative cis-interface slightly different from the one found in the strand models. For the orientation of the claudin ring in the membrane plane, we used the membrane from the strand model [52], [53]. Such modeling also required a more slanted conformation of the regions T26-L63 and Y127-F319 from domain II and III while maintaining the structural integrity of the other CPE elements. In addition, the smaller ring diameter meant insufficient space to maintain the rather elongated CPE conformation. Thus, each cCPE was further deviated from nCPE and moved from one claudin to the neighboring claudin, and the linker (V187–A204) was modified accordingly and connected to nCPE. This resulted in tightly packed nCPE domains with cCPE domains occupying six alternating claudins, while the other six claudins were in loose contact with nCPE domains. For membrane embedment, the previously equilibrated membrane from state VIIIa-CPE_6_R_6_ was used. Lipids that clashed with the newly added claudin subunits were either removed or refined. The system was generated, relaxed, and simulated as before for 100 ns without constraints to obtain state VIIId-CPE_6_R_6_R´_6_. During the stepwise equilibration, the following residue clusters were constrained for an extended time (∼ 85 ns):

i. Tyrosine cluster in cCPE – Y306, Y310 and Y312,
ii. Claudin residues fitting inside the pocket formed by the tyrosine cluster N149, P150, and L151,
iii. Residues forming the linear cis-interface between claudins – L71, F147 and M160.

One well-equilibrated structure from the state VIIId-CPE_6_R_6_R´_6_ model simulation was used to generate a variant where the comparatively tilted claudin monomers were straightened. The system was embedded in the membrane used for the VIIId-CPE_6_R_6_R´_6_ model, and lipids clashing with the claudins were removed or refined. The system was then relaxed, equilibrated with the aforementioned constraints, and finally simulated without constraints for 100 ns to obtain state VIIIc-CPE_6_R_6_R´_6_. Coulombic interactions were calculated using the short-range cutoff method, with a 9.0 Å cutoff. The RESPA algorithm was used to integrate the bonded interactions with a timestep of 2.0 fs.

### 2.5 Analysis of the MD simulation trajectories

The MD simulation trajectories of the production runs were used for various quantitative analyses. Using the MD trajectory analysis tools in BioLuminate, key parameters such as residue–residue distances and interactions (hydrogen bonds and salt bridges) were quantified. Solvent-Accessible Surface Area (SASA) data were extracted using Visual Molecular Dynamics [54] (VMD, version 1.9.4a57, release 2022–04, https://www.ks.uiuc.edu/Research/vmd). The MDAnalysis tool (version 2.3.0) [55], [56] was used to calculate the normalized contact time of the pore-lining residues with ions. Additionally, the HOLE program [57], [58] was used to analyze and visualize the pore. The protein backbone, considered for the RMSD analyses, includes the two carbons, nitrogen, oxygen, and hydrogens of the main chain of the residues. For the calculation of the mean interaction count between CPE–CPE subunit interfaces, the mean (± SD) number of electrostatic interactions (hydrogen bonds, salt bridges) formed by D48 from one CPE subunit with S60, Y129, and K131 from the neighboring subunit was calculated over the last 50 ns of simulation.

### 2.6 In vitro assays

#### 2.6.1 CPE protein preparation

For the construction of a plasmid encoding CPE carrying a C-terminal StrepII tag (CPE-Strep) fusion protein, the previously described pTrcHis-Topo-optCPEwt was used as a template [15]. With site-directed mutagenesis using a KLD enzyme mix the bases between 4285 and 4317, including the His-tag, were removed and through Gibson Assembly a Strep-tag was inserted at base 5313, at the C-terminal end of CPE. CPE was expressed as described previously [15] with modifications mentioned in Supplementary Methods.

#### 2.6.2 GST-cCPE protein preparation

GST-cCPE was expressed and purified as described previously [38]. For details, see Supplementary Methods.

#### 2.6.3 Cell culture

HEK293 (HEK) cells were cultured in Dulbecco's Modified Eagle Medium (Sigma-Aldrich, Germany) supplemented with 10 % (v/v) fetal bovine serum, 100 U/ml penicillin and 100 μg/ml streptomycin (Sigma-Aldrich, Germany) at 37 °C in a humidified 5 % CO_2_ atmosphere.

For HEK-CLDN3-WT cells, which contain the pEGFP-N1/mCLDN3 plasmid and express mouse CLDN3 (mCLDN3) with a C-terminal YFP tag, and HEK-CLDN3-S68E cells, which instead contain the pEGFP-N1/mCLDN3-S68E plasmid and express the mCLDN3 mutant with C-terminal GFP tag, both previously characterized and described [59], 0.5 mg/ml G418 was additionally added to the medium for selection.

#### 2.6.4 Cell viability assays

Cell viability assays were performed similar as described previously [16], [60]. Details are outlined in the Supplementary Methods.

#### 2.6.5 Cellular binding assays

Cellular binding assays were performed similar as described previously [38]. Details are outlined in the Supplementary Methods.

## 3 Results

### 3.1 AlphaFold2-based prediction of CPE oligomerization

Several structures of cCPE bound to CLDN3, –4, –9, or –19 [35], [36], [37], [43], [61], [62], of claudin-free CPE [29], [30], [63], [64] and, most recently, of CPE bound to CLDN4 [44], have been solved (Figure S1). These structures, along with comparisons to other β-PFTs with known pore structures (e.g., PDB IDs 3J9C, 5JZT, 5GAQ) [65], [66], [67], strongly suggest that CPE undergoes major conformational changes during pore formation. However, these changes remain unclear because no CPE pore structures, either alone or in complex with claudins, currently exist.

**Fig. 1:**
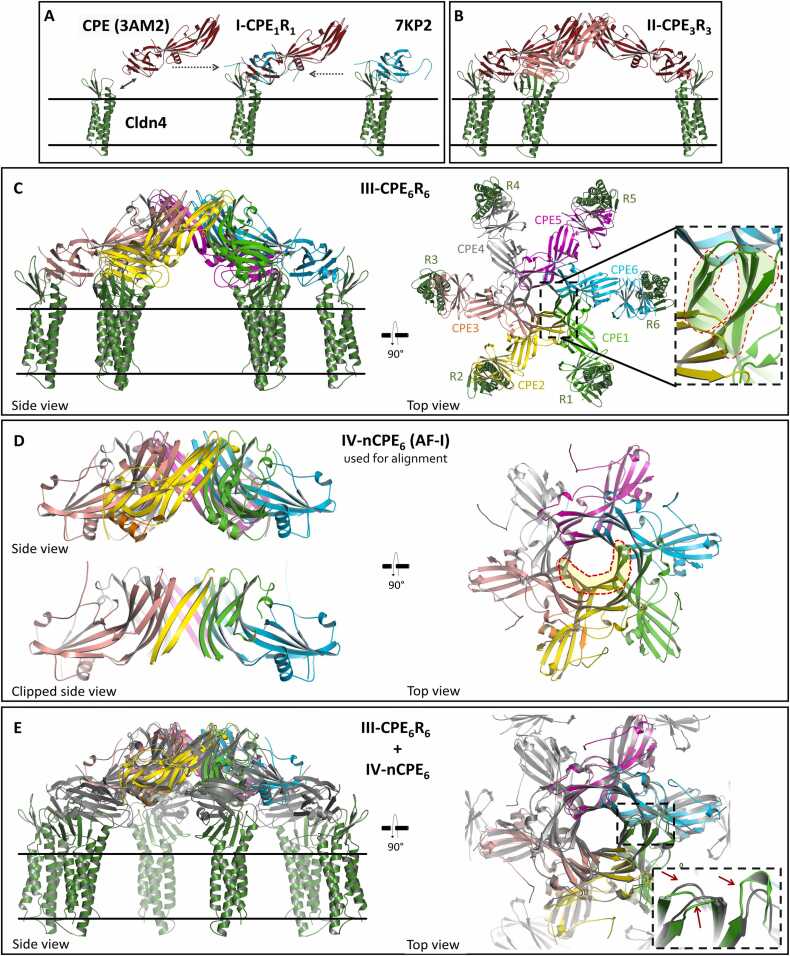
The combination of ColabFold/AlphaFold2 and structural alignment with CPE and cCPE/CLDN4 complex crystal structures (PDB 3AM2 and 7KP4) enables modeling of CPE prepore complex. (**A**) Binding of monomeric CPE to CLDN4 in the membrane (black lines) leads to state I-CPE_1_R_1_. (**B-C**) Oligomerization via state II-CPE_3_R_3_ into prepore state III-CPE_6_R_6_. Side and top views are shown. CPE (CPE1-CPE6) and claudin receptor (R1-R6) subunits are labeled. Inset: β3- and β5-strands forming the inner β-barrel (marked area, left), and β2-, -β6- and β7- strands forming the outer β-barrel (marked area, right) are shown. (**D**) State IV-nCPE_6_ corresponding to prediction AF-I (for CPE_1–202_), used as reference for alignment of the other states. Side and top views. The inner β-barrel is depicted by the clipped side view and is highlighted in the top view (dashed red line). Segment 77–111 is colored orange in one subunit (yellow cartoon). (**E**) Superposition of prepore states III-CPE_6_R_6_ (gray) and IV-nCPE_6_ (colored). Inset: Differences between IV-nCPE_6_ (green) and III-CPE_6_R_6_ (gray). These differences (red arrows) result in the absence of clashes in IV-nCPE_6_.

**Fig. 2:**
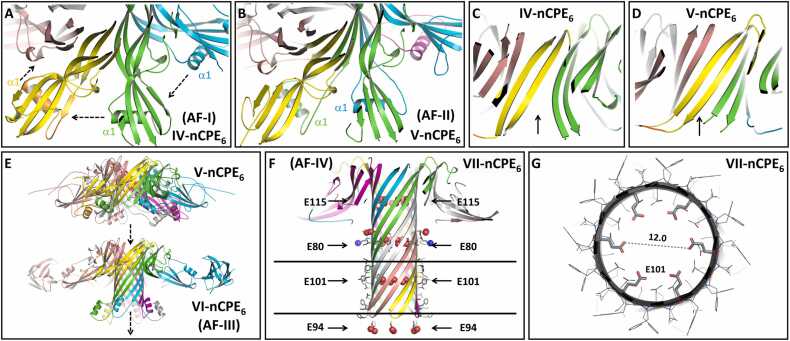
Prediction of the sequential transition from prepore to pore states of the nCPE hexamer. (**A-D**) Transition from prepore state IV-nCPE_6_ (prediction AF-I, for CPE_1–202_) to prepore state V-nCPE_6_ (prediction AF-II, for CPE_1–202_). See Fig. 1D for IV-nCPE_6_ overview. (**A, B**) The direction of movement/swapping of the α1-region between CPE subunits is indicated by arrows in A. The segment 77–111 including α1 is highlighted for one subunit in orange. (**C, D**) Completion of the β-barrel, as indicated by arrows. (**E, F**) Side views showing stepwise β-barrel extension in prepore states V-nCPE_6_ (AF-II), VI-nCPE_6_ (AF-III, for CPE_26–319_) and pore state VII-nCPE_6_ (AF-IV for CPE_1–202_), representing potential intermediates and the final pore. (**F**) Clipped side view of the extended β-barrel. Three hexa-glutamate rings (E80, E101 and E115) forming constrictions along the β-barrel pore are indicated by arrows. The hexa-glutamate ring formed by E94 at the intracellular opening of the pore is also shown. Negatively (red) and positively (blue) charged side chains are shown as spheres, hydrophobic residues facing lipids are shown as sticks, membrane boundaries are indicated as black lines. (**G**) Clipped top view on ring formed by E101 in the middle of the transmembrane region. The six E101 are shown as sticks, other residues as lines, and the distance (12 Å) between two opposing E101 O-atoms as a dotted line.

Given the advances in the prediction of protein complexes by AlphaFold2, we used its variant ColabFold v1.5.5 [45], [68] to predict CPE homo-oligomers. Several input parameters, including full-length CPE (1−319) and truncated sequences (1−202, 26−202, 26−319), were used (see Methods Section 2.2 for details). Only prediction results consistent with key experimental findings were considered, specifically hexa- or heptameric ring-shaped architectures with a central pore formed by nCPE. Initially, the most meaningful results were obtained with CPE_1–202_ (nCPE), which lacks the claudin-binding domain (cCPE, residues 203–319). We conclude that the removal of cCPE in the prediction suppressed the overcontribution of non-pore-forming folds from monomeric CPE structures and cCPE/claudin complex structures (PDB IDs listed above) in the AlphaFold2 training datasets. Some of the output CPE oligomer structures (e.g., AlphaFold2/ColabFold (AF) prediction I, AF-I, Fig. 1) reproduced the fold of nCPE found in CPE monomer crystal structures (PDBs 2XH6, 3AM2, 3ZIX, 8U5F, Figure S1), while others showed a different conformation for the region 70–116 (AF-II, AF-III, and AF-IV, Fig. 2). Importantly, all these predicted structures contained a central pore-shaped β-barrel. Strikingly, the length of the barrels increased from AF-I to AF-IV (34 Å, 38 Å, 51 Å, and 81 Å, respectively), suggesting subsequent conformational states converting the initial, receptor-unbound monomeric CPE fold (PDB ID 3AM2) via prepore intermediates (AF-I–AF-III) into the functional membrane-permeating pore (AF-IV). Thus, we proceeded with a step-by-step analysis of the AlphaFold2/ColabFold predictions AF-I to AF-IV.

### 3.2 Decoding CPE Pore Formation: Insights from alignment of AlphaFold2-predicted oligomers with solved CPE and cCPE/CLDN4 structures

To connect the selected AlphaFold2 predictions with the experimentally solved CPE/claudin structures, we first aligned the crystal structures with the predicted structure that had the highest segment overlap, which is AF-I (Fig. 1D). This alignment allowed us to develop a sequential model of CPE/claudin complex assembly (Fig. 1) and pore formation (Fig. 2).

Initially, monomeric CPE binds to a receptor, such as CLDN4, in the cell membrane (Fig. 1A). The conformation of this first CPE/claudin complex was generated by aligning the structure of CPE (PDB ID 3AM2) with a complex structure of cCPE bound to human CLDN4 (PDB ID 7KP4), and a membrane embedment prediction for CLDN4 (see methods Section 2.3). This complex state is referred to as I-CPE_1_R_1_, as we use the following nomenclature here and for subsequent states: X-CPE_i_R_j_, with X (roman numeral) for the specific state of the sequential assembly process, i for number of CPE subunits and j for number of receptor (i.e., CLDN4) subunits in the complex.

According to gel shift analysis and other biochemical data [41], six CPE/CLDN4 complexes (I-CPE_1_R_1_) oligomerize via nCPE. Consequently, we assumed intermediate states, such as state II-CPE_3_R_3_ (Fig. 1B) and state III-CPE_6_R_6_ (Fig. 1C). State III-CPE_6_R_6_ exhibits cyclic symmetry and contains a short β-barrel-like pore formed by two β-strands (residues 68–77 (β3) and 111–120 (β5)) from each of the six CPE subunits in the center, surrounded by a second, looser β-barrel formed by three additional β-strands of the CPE subunits (residues 57–63 (β2), 127–135 (β6), and 158–167 (β7) (Fig. 1C, inset). This architecture resembles the central cap of the mushroom-like cap-and-stem architecture seen in other β-PFT pores (e.g., aerolysin and lysenin, with PDB IDs 5JZT, and 5GAQ, respectively [66], [67]). Importantly, the nCPE segments of state III-CPE_6_R_6_ align well with the nCPE subunits of the AlphaFold2-prediction AF-I (Cα RMSD of ∼ 1.9 Å, Fig. 1D, E). The main difference between nCPE in AF-I and in state III-CPE_6_R_6_ is the presence of minor steric clashes in III-CPE_6_R_6_ (Fig. 1E inset), which result from the structural alignment of six rigid CPE subunits with AF-I. These clashes are absent in the prediction AF-I that is also referred to as state IV-nCPE_6_ (IV since it follows state III, nCPE since it lacks the cCPE domain and receptors). State IV-nCPE_6_ likely represents a CPE prepore structure formed by initial CPE oligomerization and is not yet penetrating the membrane, similar to what is shown for state III-CPE_6_R_6_ in Fig. 1C. Up to this point, alignment of the predicted nCPE oligomer AF-I with CLDN4 and CPE crystal structures led to a prepore model with plausible architecture and orientation toward the membrane.

Next, we investigated conformational changes during the transition from a prepore into a pore. Given the high structural similarity between cCPE in monomeric CPE and in cCPE/claudin crystal structures (Figure S1B, [37]), we assumed that mainly nCPE undergoes changes during the prepore-to-pore transition. Consequently, at this point, we focused on additional AlphaFold2 predictions of the nCPE hexamer without considering cCPE and the receptors (claudins). Interestingly, the prediction AF-II (referred to as state V-nCPE_6_) is very similar to AF-I (state IV-nCPE_6_), with the difference that it contains a notable domain swap of the amphipathic α-helix segment α1 (residues 92–105) in domain II (Figure S1A). In prepore state IV-nCPE_6_, this segment interacts with the β-sheet (β1, β4, β6, β7, β8) in the CPE domain II (Figure S1A) of the same subunit. However, in prepore state V-nCPE_6_, α-helix segment α1 is swapped into the corresponding β-sheet of the neighboring CPE subunit (Fig. 2A, B, Movie S1). This swap closes a gap in the inner β-barrel lining the prepore (Fig. 2C, D). In the next modeled state VI-nCPE_6_ (AF-III), the interaction of the α1-segment (92−105) with the β-sheet in CPE domain II is lost, and the adjacent segments (residues 77–83 and 106–111) extend the central β-barrel, resulting in its elongation from 38 Å to 51 Å (Fig. 2E). Finally, the region 84–105 stretches into a β-hairpin and extends the β-barrel to the full stem (81 Å) of the mushroom-like architecture and forms the membrane-spanning region (Fig. 2F, state VII-nCPE_6_, Movie S2**,** S3). Strikingly, in region 81–108, hydrophobic residues point outward while hydrophilic residues point toward the pore lumen, making this the proposed transmembrane region. Beyond this region, hydrophilic residues face outward. Along the β-barrel pore lumen, three hexa-glutamate rings are formed by E101, E80, and E115, respectively. The O-atoms of the glutamate residues at the narrowest ring are ∼ 12 Å apart from each other (Fig. 2F, G). Therefore, the pore lining fits perfectly as a cation-selective transmembrane pore formed by CPE. This brings the modeled state VII-nCPE_6_ as the active pore and the pore assembly intermediates (Fig. 1, Fig. 2) in agreement with conductivity measurements and cell viability experiments [69], [70].

### 3.3 Comparison of hexamer and heptamer pore models of nCPE with structures of other β-pore forming toxins

Monomeric CPE shows structural homology to *A. hydrophila* aerolysin that forms a heptameric pore [29], [30]. Hence, we also predicted nCPE heptamers with AlphaFold2. Some of these predictions (e.g., AF-VI) resulted in a heptameric pore with a similar stem and cap center architecture as that of the aerolysin pore (Figure S2). However, when compared to the nCPE hexamer model (VII-nCPE_6_), the heptameric pore (VII-nCPE_7_) had the following inconsistencies: (i) The β-hairpin tip was irregular, and the length of the pore barrel was too short for the heptamer to fully span through the membrane (71 Å for heptamer instead of 81 Å for hexamer, Figure S2A, B); (ii) the hydrophilic Q97 that points toward the pore lumen in the hexamer points toward the hydrophobic lipids surrounding the pore in the heptamer. Conversely, the opposite is true for hydrophobic I96 (Figure S2A, B). For the heptamer, these residue orientations would be energetically unfavorable. Due to these observations and the biochemical data supporting a CPE hexamer [41], the heptamer model was not analyzed further.

**Fig. 3:**
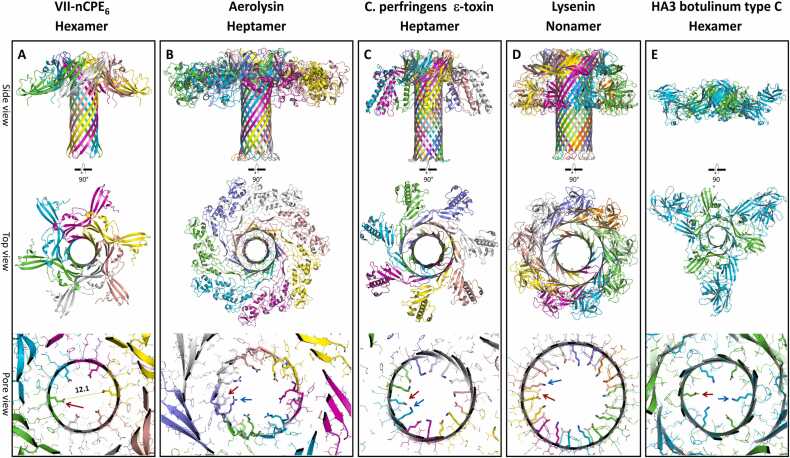
Comparison of the (**A**) nCPE hexamer model (state VII-nCPE_6_, AF-IV) with the β-pore forming toxins (**B**) aerolysin, PDB 5JZT, homo-heptamer, (**C**) *C. perfringens* ε-toxin, PDB 6RB9, homo-heptamer, (**D**) lysenin, PDB 5GAQ, homo-nonamer, and (E) the HA3 subcomponent of *C*. *botulinum* type C progenitor toxin, PDB 2ZS6, hetero-hexamer. Side view, top view and close-up of pores in cap region of the toxins are shown. Charged residues are shown as sticks (red and blue arrows for negative and positive charges, respectively) and other residues as lines.

Comparison of the nCPE hexamer pore model (state VII-nCPE_6_) with the structures of the other β-PFTs *A. hydrophila* aerolysin, *C. perfringens* epsilon toxin (ETX) and *E. fetida* lysenin showed that all these toxins share a similar mushroom-like architecture with a β-barrel pore stem and two concentric β-barrels in the center of the cap region (Fig. 3). Aerolysin and ETX form heptameric pores, lysenin forms nonameric pores, and CPE forms hexameric pores. The nCPE model shares its hexameric architecture, as well as structure and sequence homology, with the HA3 subcomponent of the *C*. *botulinum* type C progenitor toxin (sequence identity: 27 % between CPE_1–200_ and HA3a_8–184_, and 25 % between CPE_1–319_ and two of three domains in HA3b) [29]. The corresponding crystal structure (Fig. 3E; PDB ID 2ZS6 [71]) was one of the templates used by AlphaFold2 for the predictions (see methods 2.2). Similar to the other toxins, HA3 contains two concentric β-barrels surrounding the central pore. In contrast to the CPE pore model (VII-nCPE_6_), the HA3 pore is of insufficient length to cross the membrane, thereby likely resembling an inactive prepore state (Fig. 3E). Consistently, the HA3 oligomer shows most structural similarity with the prepore state IV-nCPE_6_, with which it also shares the 5-stranded β sheet (β1, β4, β6, β7, β8) and the interacting α-helix (α1 of the CPE domain II (Figs. 1D, 3A, E, S1A).

The diameters of the different toxins increase with the number of subunits. In addition, the pore linings differ significantly. CPE has numerous negatively charged, but no positively charged residues in the pore lumen. In contrast, the pores of other toxins contain both (Fig. 3, pore views). This charge distribution fits to the CPE-specific cation selectivity, which is not observed for the other β-PFTs [70]. In summary, the observed similarities and differences between the presented experimental structures and the hexameric nCPE pore model support the plausibility of the latter, which represents the first described homo-hexameric β-PFT pore model.

### 3.4 MD simulations support the predicted hexameric mushroom-like architecture of the nCPE pore

The pore model state VII-nCPE_6_, was further analyzed with respect to stability and ion permeability using molecular dynamics (MD) simulations. For this, a workflow based on Schrödinger´s Maestro- and Desmond platform was used, as previously established for the simulation of claudin-based paracellular ion channels [52], [53]. The protein complex VII-nCPE_6_ as shown in Fig. 3A was embedded in POPC lipids, equilibrated, and simulated without constraints for 100 ns.

Throughout the simulation, the whole stem and cap structure of the complex including the two concentric β-barrel elements in the cap region remained stable (Figure S3A, Movie S4). The proposed transmembrane part of the stem (residues 80–108) remained embedded in the membrane, with the β-hairpins including the negatively charged tip (E94) extending to the cytoplasmic side (Figure S3B). Moreover, the water- and ion-filled pore barrel remained stable (Figure S3B, C). Preservation of structural architecture throughout the simulation is reflected by a mean protein backbone RMSD for the last 50 ns of the simulation of ∼ 3.85 Å relative to the initial structure. The three hexa-glutamate rings formed along the pore by E101, E80, and E115, respectively, interacted strongly with Na^+^ ions (Figure S3B-E). Only very few Cl^-^ ions were observed within the pore. Thus, the simulation underlined the known cation selectivity of CPE [22], [23], [24] and further supported our pore model.

### 3.5 Anchoring of the CPE β-barrel pore to the receptors and rearrangements to span the pore through the membrane

**Fig. 4:**
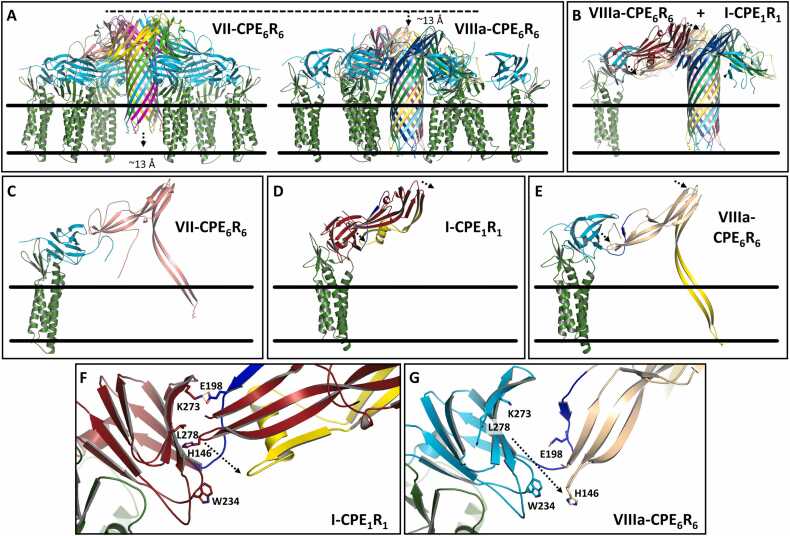
Conformational change in linker region between cCPE and nCPE (residues 193–205, blue) allowing full transmembrane penetration of the β-hairpin tips of the pore. (**A**) Comparison of CLDN4-bound CPE hexamer pore complexes without (state VII-CPE_6_R_6_) and with downwards shift of nCPE (state VIIIa-CPE_6_R_6_). E94 at β-hairpin tip is shown as stick. (**B**) Clipped view of state VIIIa-CPE_6_R_6_ focused on one claudin with superimposed state I-CPE_1_R_1_ to visualize the conformational difference between the monomeric CPE structure (PDB ID 3AM2 in I-CPE_1_R_1_, red) and CPE in VIIIa-CPE_6_R_6_ (cyan, beige). Individual CPE-claudin dimers are shown separately for VII-CPE_6_R_6_ (**C**), I-CPE_1_R_1_ (**D**) and VIIIa-CPE_6_R_6_ (**E**). The region 73–116 that changes conformation is labeled yellow. In I-CPE_1_R_1_ it includes the α1-helix, in VII-CPE_6_R_6_ and VIIIa-CPE_6_R_6_ it forms the main part of the pore β-barrel. The manual shift of nCPE in VIIIa-CPE_6_R_6_ relative to the position in I-CPE_1_R_1_is labeled by dashed arrows in (B, D-G). (**F, G**) Close-ups of (D, E) highlighting the move of H146 at tip of nCPE from vicinity to L278 of cCPE in I-CPE_1_R_1_ toward W234 of cCPE in VIIIa-CPE_6_R_6_.

We next examined the anchoring of the hexameric CPE pore to the claudin receptors embedded in the membrane. For this, stepwise alignments were carried out to position VII-nCPE_6_ on six cCPE/CLDN4 complexes: (i) IV-nCPE_6_ and six cCPE-CLDN4 (7KP4) with III-CPE_6_R_6_; (ii) VII-nCPE_6_ with IV-nCPE_6_; (iii) Removal of IV-nCPE_6_ and III-CPE_6_R_6_. This resulted in state VII-nCPE_6_R_6_. Prediction of the membrane plane of the claudins in this complex using the PPM server [46] showed that the tips of the β-hairpins of the pore did not entirely span through the membrane (∼ 13 Å missing, Fig. 4A, VII-nCPE_6_R_6_). We speculated that a conformational change in the linker region between cCPE and nCPE (residues K193-A205, the blue segment in Fig. 4D-G) would facilitate membrane permeation of the pore. Our predicted pore structure VII-nCPE_6_R_6_ did not yet contain a linker between nCPE (from VII-nCPE_6_) and cCPE (from 7KP4) (Fig. 4C). For linkage and to move nCPE but not cCPE toward the membrane as a rigid body, most of the linker region (K197-A205) was manually joined to cCPE. For this, H146 at the tip of nCPE was moved from L278 at the upper rim of cCPE to the lower rim close to W234 of cCPE (Fig. 4B, D-G, black dashed arrows). This resulted in a full-length CPE hexamer pore (state VIIIa-CPE_6_R_6_), in which, unlike as in state VII-nCPE_6_R_6_, the β-hairpin tips of the pore span through the entire membrane (Fig. 4A, B, E).

We also tested another cCPE-nCPE linker variant (state VIIIb-CPE_6_R_6_) based on an AlphaFold2 prediction for CPE_26–319_ (AF-III). Similar as for state VIIIa-CPE_6_R_6_, nCPE was shifted downwards so that H146 of nCPE was close to P233 at the lower rim of cCPE (Figure S4F-J, black dashed arrow). Moreover, for this pore state (VIIIb-CPE_6_R_6_), the β-hairpins of the pore reached the cytoplasmic side of the membrane (Figure S4F-J). However, here the region P191-A205 (blue in Fig. 4D-G, S4H-J) shows an α-helix, which would imply a rather unlikely β-to-α transition compared to the state III-CPE_6_R_6_ (containing CPE, PDB ID 3AM2). Thus, we carried out further analysis on state VIIIa-CPE_6_R_6_.

### 3.6 MD simulations support the model of the receptor-anchored and membrane-spanning hexameric CPE cation pore

**Fig. 5:**
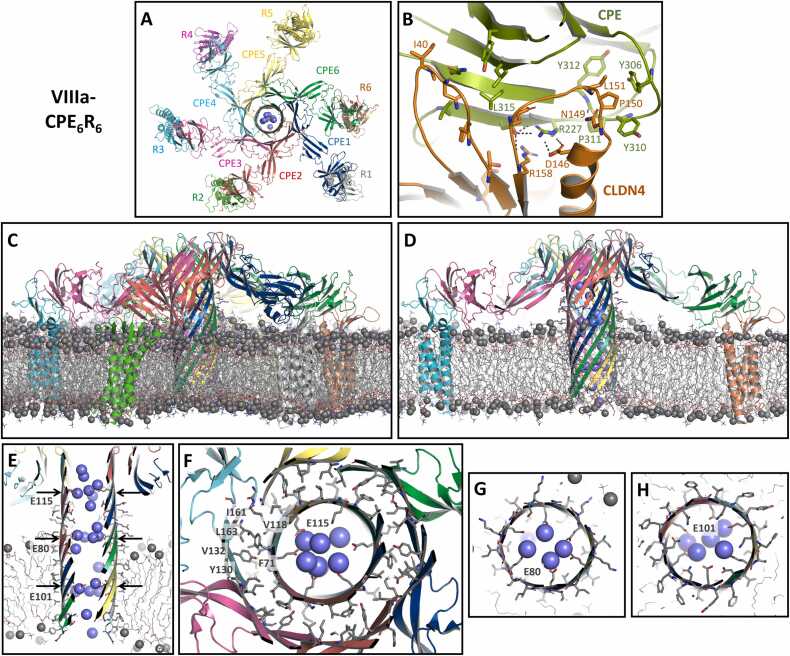
MD simulation of the CPE/CLDN4 pore complex state VIIIa-CPE_6_R_6_. Snapshots after 100 ns of free simulation are shown with protein as cartoon, relevant residues as sticks, lipid acyl chains as gray lines, phosphate head groups as gray spheres, sodium ions as blue. (**A**) VIIIa-CPE_6_R_6_ shown as top view with labeled CPE (CPE1-CPE6) and CLDN4 receptor (R1-R6) subunits. (**B**) Details of the stable interaction between cCPE domain and CLDN4. Key residues are labeled and key electrostatic interaction shown as dashed lines. (**C, D**) VIIIa-CPE_6_R_6_ shown as side view (clipped in (D)). The pore barrel and the pore cap are well preserved. The pore complex is well embedded in the membrane. (**E**) Clipped side view of pore to illustrate membrane embedding and strong presence of sodium ions in the pore lumen. (**F**) The inner and outer β-barrel in the cap region (upper arrow in (E)) are held together mainly by hydrophobic interactions. Some key residues are labeled. In the center, a ring of six E115 residues strongly attracts cations (spheres). Rings formed by six E80 residues (**G**) slightly above membrane plane (middle arrow in (E)) and by six E101 residues (lower arrow in (E)) within the membrane plane (**H**) also strongly attract cations.

The model of state VIIIa-CPE_6_R_6_ of the pore complex was further analyzed with respect to stability and ion permeability using MD simulations. The protein complex VIIIa-CPE_6_R_6_ as shown in Fig. 4 was embedded in POPC lipids, equilibrated, and simulated without constraints for 100 ns. The transmembrane regions of both the CPE hexamer (residues 80–108) and the six claudins remained well embedded in the membrane, and the whole complex remained stable throughout the simulation, with a mean protein backbone RMSD of 4.57 ± 0.37 Å for the last 50 ns of simulation with respect to the initial structure (Fig. 5, Movie S5). For CPE, this included the β-hairpins, their cytoplasmic tips with E94, the inside and outside of the stem barrel, and the concentric β-barrel element in the cap region (Fig. 5A, C-E). In addition, the interaction between cCPE and CLDN4 remained stable throughout the simulation, including the interfaces observed in the experimental cCPE/CLDN4 structures (5B2G, 7KP4, 8U4V [36], [72]). In particular, L151 in the ECS2 of CLDN4 was stably inserted into the triple tyrosine pocket formed by Y306, Y310, and Y312 of cCPE (Fig. 5B, [34], [62], [73]). The cap region was stabilized by the two concentric β-barrels, in which the intramolecular contact between two β-sheets is maintained mainly by hydrophobic interactions between V58, L63, F71 and V118 of the inner barrel and T120, V128, Y130, V132, I161, L163 of the outer barrel (Fig. 5F), similar as in the monomeric CPE (PDB ID 3AM2).

Inter-subunit interactions are mediated by H-bonds between neighboring β-strands and by electrostatic side chain interactions (e.g., E67-K165). The three hexa-glutamate rings formed along the pore by residues E101, E80, E115, respectively, interacted strongly with Na^+^ ions (Fig. 5E-H). Similar results were obtained for the respective simulation of CPE hexamer state VIIIb-CPE_6_R_6_ (Figure S5), indicating that the conformation of the linker region has no influence on the pore lumen. In summary, the MD simulations strongly support the modeled architecture, claudin anchoring, membrane embedment, pore lining, and cation selectivity of the CPE hexamer pore.

### 3.7 Do claudins form a dodecameric ring during assembly of the CPE pore complex?

**Fig. 6:**
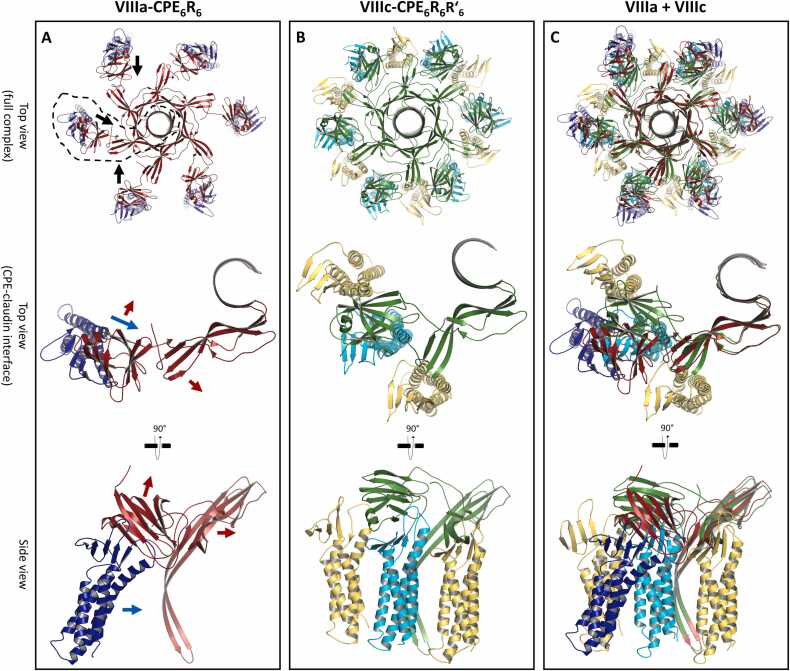
Modification of relative cCPE/nCPE positions for modeling of state VIIIc-CPE_6_R_6_R´_6_. (**A**) State VIIIa-CPE_6_R_6_: CPE pore hexamer (red) bound to six individual claudins (blue). (**B**) State VIIIc-CPE_6_R_6_R´_6_: CPE pore hexamer (green) bound to a dodecameric claudin ring. Claudin subunits (R) primarily bound to CPE are colored cyan, and claudin subunits (R´) serving as secondary receptors are colored yellow. (**C**) Superposition of VIIIa-CPE_6_R_6_ and VIIIc-CPE_6_R_6_R´_6_. Top views of complex (top), single CPE subunits of the complex bound to claudins in top (middle) and side view (bottom). Arrows indicate direction of movement for cCPE (red) and claudin (blue) as they transition from state VIIIa-CPE_6_R_6_ to state VIIIc-CPE_6_R_6_R´_6_. 100 ns snapshots of MD simulations are shown (see Fig. 5 and below in 3.8).

Biochemical data suggest that the CPE pore complex contains more claudin than CPE subunits [41]. Furthermore, low-affinity CPE receptors, such as CLDN1 or CLDN5, have been detected in CPE/claudin complexes along with high-affinity CPE-receptors like CLDN4 and CLDN3 [41], [74]. Molecular modeling [34] predicted and crystal structures [36] verified a 1:1 stoichiometry of the high-affinity cCPE-claudin interaction. In contrast, for the CPE/claudin complex, a 1:2 stoichiometry was suggested [41], [75]. Thus, either only one subunit of a claudin dimer directly interacts with CPE, or the second one is in contact with CPE via additional low-affinity interaction sites. For the second claudin within the complex, different binding configurations are conceivable. However, due to the polymerization capability of claudins, we speculated that 12 claudin subunits could form a dodecameric ring via a bent variation of the linear cis-interaction typically found in claudin polymer-based tight junction strands [14], [52], [76], [77], [78]. It has been shown that binding of cCPE to claudins alters the conformation of the claudin regions involved in the linear cis-interaction [43], [60]. In addition, it was suggested that differences in these interface regions can affect the rotational angle or relative positioning of neighboring claudin subunits [43], [53], [78]. Thus, it is plausible that cCPE binding induces bending of up to ∼ 30° in the typically straight cis-interface, resulting in a 360° rotation for a dodecamer and the formation of a claudin ring. This putative ring was modeled based on a linear cis-interface in a claudin strand (Figure S6H) and 30° rotations between the subunits (Fig. 6, Figure S6 and see methods 2.4.2). The resulting ring had a smaller diameter than the discontinuous ring formed by six claudins in the complex state VIIIa-CPE_6_R_6_ (∼ 148 Å vs ∼ 182 Å for the S58-S58 Cα distance, Fig. 6C, top). Consequently, for docking CPE to the ring, the straight connection between nCPE and cCPE in state VIIIa-CPE_6_R_6_ had to be changed to an angled one, to keep the structural integrity of both the nCPE hexamer and the cCPE/CLDN4 complex (Fig. 6A, B, middle). This resulted in a tightly packed complex (state VIIIc-CPE_6_R_6_R´_6_) without steric conflicts, in which every second CLDN4 subunit (referred to as R for primary receptor) interacted with CPE as in state VIIIa-CPE_6_R_6_. The other claudin ring subunits (referred to as R´ for secondary receptor) were in loose contact on one side with cCPE and on the other side with the domain II of nCPE (Fig. 6B).

**Fig. 7:**
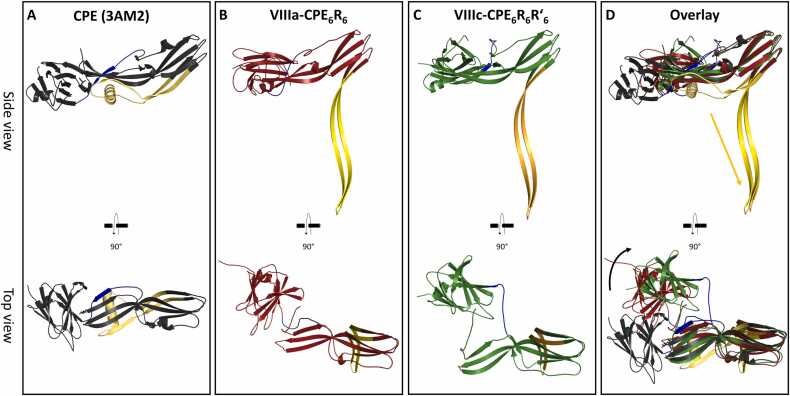
Comparison of the CPE monomer crystal structure (**A**; top, side view, bottom, top view) with state VIIIa-CPE_6_R_6_ (**B**), state VIIIc-CPE_6_R_6_R´_6_ (**C**). In (**D**), a superposition of the three structures is shown. Conformational changes: Yellow: residues 70–116, region forming the pore barrel (straight yellow arrow); blue: residues 191–204, linker between cCPE and nCPE. Note the shift and turn of cCPE relative to nCPE between states (curved black arrow). For VIIIa-CPE_6_R_6_ and VIIIc-CPE_6_R_6_R´_6_, snapshots after 100 ns MD simulation are shown (see Fig. 5 and in 3.8).

The complete conformational change of a CPE subunit from the monomeric state to the two alternative pore states VIIIa-CPE_6_R_6_ and VIIIc-CPE_6_R_6_R´_6_ are depicted in Fig. 7. Since the precise tilt of the claudins in the membrane plane is unclear, we also generated a variation of state VIIIc-CPE_6_R_6_R´_6_ (VIIId-CPE_6_R_6_R´_6_) in which the tilt of the claudins was slightly different. Details of this variant are shown in Figure S7.

### 3.8 MD simulations support a cation-selective CPE pore anchored to a dodecameric claudin ring

**Fig. 8:**
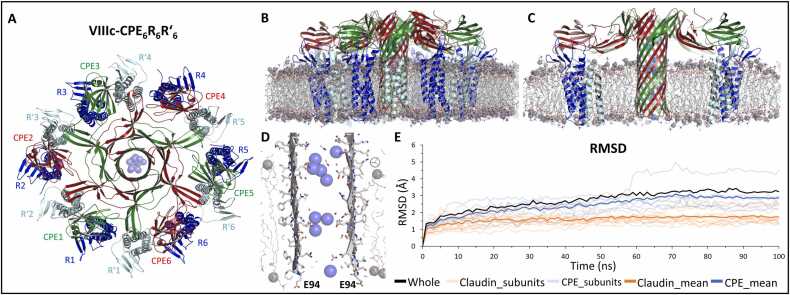
MD simulation of CPE pore complex state VIIIc-CPE_6_R_6_R´_6_. Snapshots are shown after 100 ns of simulation, with the protein as cartoon, relevant residues as sticks, lipid acyl chains as gray lines, phosphate headgroups as gray spheres, and sodium ions as blue spheres. (**A**) Top view with CPE (CPE1-CPE6, alternating green and red), primary receptor CLDN4 (R1-R6, blue) and secondary receptor CLDN4 (R´1-R´6, cyan) subunits. (**B, C**) Side view (clipped in (C)). The pore β-barrel and cap are well preserved throughout the simulation. The CPE transmembrane region and the bound CLDN4 are well embedded in the membrane. (**D**) Clipped side view of membrane-spanning pore region illustrating hydrophobic residues facing lipids while hydrophilic ones line the pore lumen. (**E**) Root-mean-square deviation (RMSD) of protein backbone over the simulation time (100 ns) with respect to the starting structure (0 ns) is plotted. RMSD for whole complex reached plateau at ∼ 3.0 Å after ∼ 50 ns. In addition, RMSD for individual subunits and mean RMSD for six CPE and six claudins are shown.

To test the stability of the two modeled complexes VIIIc-CPE_6_R_6_R´_6_ and VIIId-CPE_6_R_6_R´_6_ with the different linker conformations within a membrane, we carried out MD simulations as described above (3.5, 3.7). Both complexes exhibited similar stability concerning the pore stem, the cap region, the main cCPE-claudin interaction site, membrane spanning, pore lining, and cation attraction (Fig. 8, S8, S9, Movie S6), comparable to the simulations of the complex states VII-nCPE_6_ and VIIIa-CPE_6_R_6_ (Figs. 5, S3). Since the state with the CPE pore within a dodecameric CLDN4 ring is the most informative and complete, all further analyses of the MD simulations were based on this state. Furthermore, for VIIIc-CPE_6_R_6_R´_6__,_ the tilt of the claudins within the membrane was more consistent with the tilt observed in tight junction strand and channel models [52], [53], [77] than for the variant with the other linker conformation (VIIId-CPE_6_R_6_R´_6_). Thus, state VIIIc-CPE_6_R_6_R´_6_ was chosen for further analysis.

Throughout the 100 ns simulation run, the central, 83 Å long pore β-barrel remained stable with extended H-bonds between the twelve β-strands (Fig. 8A-C, Movie S6). All six β-hairpin tips with E94 consistently extended to the cytoplasmic side (Fig. 8D). Hydrophobic residues on the lipid-facing side of the β-barrel transmembrane region (80−108) remained well embedded in the membrane, whereas the pore lumen was lined with hydrophilic, non-charged, and the negatively charged residues E80, E101, E115. The pore lumen remained filled with water and sodium ions, but not with chloride ions during the simulation (Fig. 8A-D). The overall stability of the complex was reflected by a root-mean-square deviation (RMSD) of the protein backbone reaching saturation at ∼3.0 Å after ∼50 ns (Fig. 8E). Notably, the claudins fluctuated around ∼1.5 Å, which is comparable to the RMSDs of claudins in tight junction strand models [52], [53]. A detailed analysis of the RMSDs and RMSFs is shown in Figure S9.

### 3.9 The ion permeation pathway of the CPE pore

**Fig. 9:**
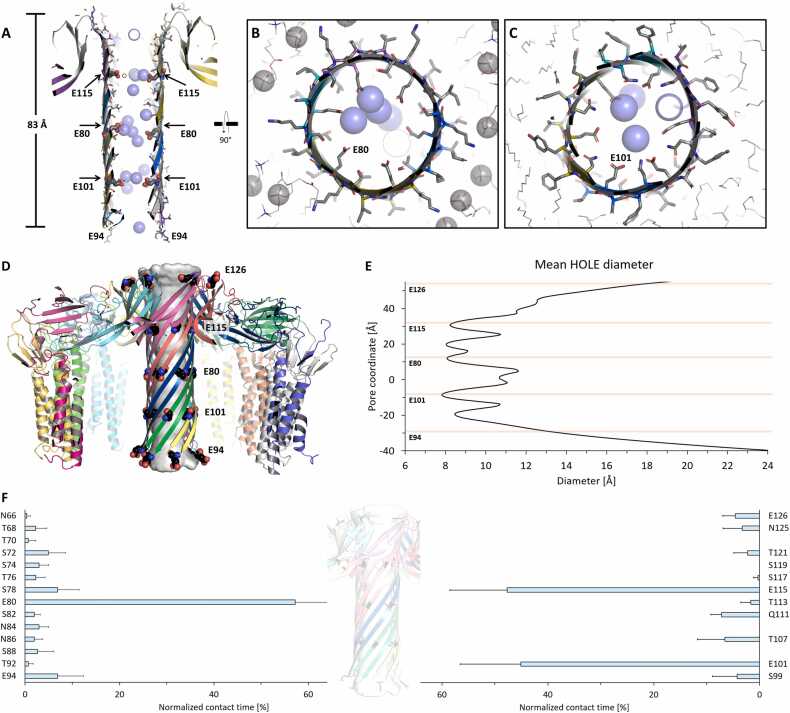
Pore diameter and interaction of pore-lining residues with sodium ions in state VIIIc-CPE_6_R_6_R´_6_. Snapshots after 100 ns of simulation are shown. (**A**) Clipped side view of pore barrel to illustrate pore lining, strong presence of sodium ions and position of hexa-glutamate rings (arrows). (**B**) Ring formed by six E80 residues slightly above membrane plane. (**C**) Ring formed by six E101 residues within the membrane plane. (**D**) Pore path and dimensions (gray surface) of the pore β-barrel, as obtained by quantitative analysis of simulation trajectory data using HOLE [58]. CPE and CLDN4 subunits are shown as colored cartoons. The positions of pore-lining glutamates are shown as spheres in the cartoon. (**E**) Diameter along the pore coordinates calculated by HOLE. Constrictions of the pore diameter near the rings formed by E80, E101, and E115 were observed. Mean minimal pore diameter at E101: 7.8 Å. (**F**) Normalized contact times of pore-lining residues with Na^+^ ions over simulation time (last 50 ns). E80, E101 and E115 interacted more (> 40 % of time) than neighboring polar residues, as well as E94 and E126 residing at the pore periphery (all ∼ 5–10 % of time).

We then analyzed the ion permeation pathway of the CPE pore in state VIIIc-CPE_6_R_6_R´_6_ in more detail. Along the pore barrel, three hexa-glutamate rings were formed by E80, E101, and E115, respectively, which strongly attracted sodium ions (Fig. 9A-C). The strong cation attraction in our simulations matches the experimentally demonstrated high cation selectivity of the CPE pore [69], [70]. The pore lumen of the simulation trajectory was analyzed using HOLE [58]. In the mean pore diameter profile, we observed pronounced constrictions of the pore at the rings formed by E80, E101, and E115, respectively. In particular, the narrowest constriction, with a mean diameter of 7.8 Å, was observed at the E101 ring, which is the one closest to the cytoplasmic side (Fig. 9D, E).

To investigate the interaction of pore-lining residues with incoming Na^+^ ions, we calculated the normalized contact time of these residues with ions within the simulation time. The ring-forming glutamates E80, E101, and E115 exhibited the longest contact times, each interacting with Na^+^ ions for more than 40 % of the simulation duration (Fig. 9F). Adjacent polar residues on either side of the ring glutamates (Fig. 9F) also showed slightly prolonged contact times of 5–10 % of the simulation duration, suggesting that these residues likely play supporting roles in ion passage. In contrast to E80, E101, and E115, which are located inside the pore, E94 and E126, which are located at the periphery of the pore, showed short contact times (∼5–10 %). These two glutamates may contribute to cation attraction toward the pore barrel.

### 3.10 Detailed analysis of subunit interfaces in the pore complex state VIIIc-CPE_6_R_6_R´_6_

**Fig. 10:**
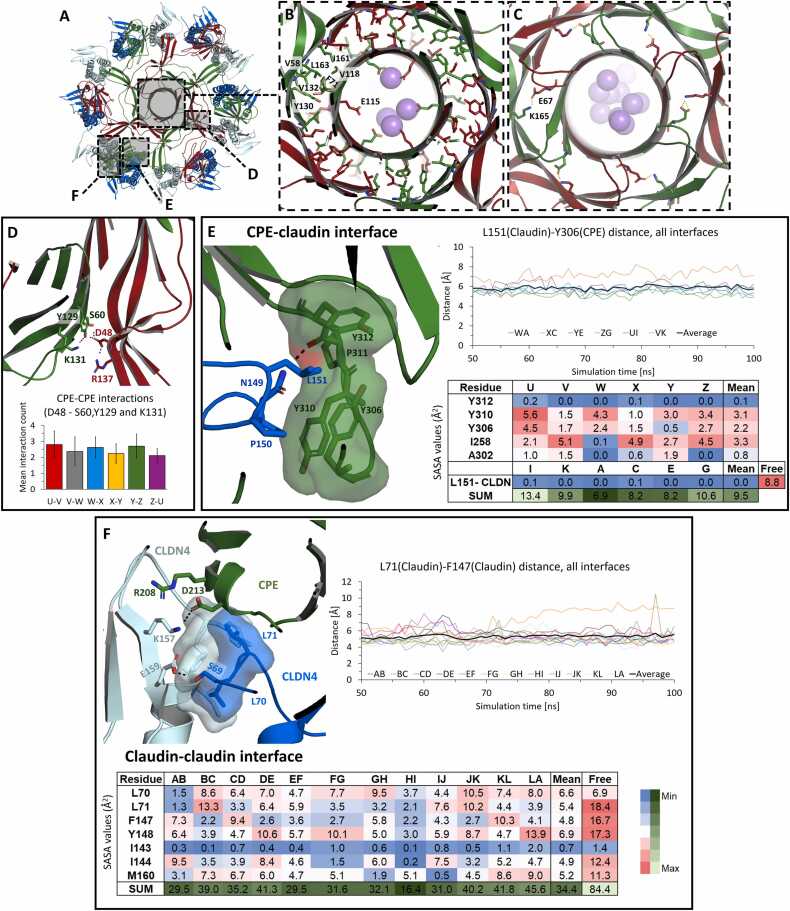
Analysis of intra- and inter-subunit interfaces after MD simulation of the CPE pore complex state VIIIc-CPE_6_R_6_R´_6_. Structure images show snapshots after 100 ns. (**A**) Top view of the complex with CPE (alternating green and red), primary receptor CLDN4 (blue), and additional secondary CLDN4 receptor subunits (cyan). Close-up regions shown in (B-F) are highlighted. (**B**) Interactions within the cap region of a CPE subunit. The inner and outer β-barrels are held together primarily by hydrophobic interactions. Key residues are shown. (**C**) CPE-CPE interactions in the cap region of the pore. The E67-K165 interaction between inner and outer β-barrel is shown. (**D**) Top: Electrostatic network at the backside of the bottom of the outer β-barrel. Bottom: Mean interaction count ( ± SD) for D48 with S60, Y129, and K131 within the last 50 ns of simulation for the six CPE-CPE subunit interfaces. (**E**) Key CPE-CLDN4 interface. Left: Cartoon of the interface, key residues are shown as sticks, and the binding pocket in CPE as semi-transparent surface. The perspective is turned by ∼ 180° with respect to (A). Top: Distance between L151 (Cγ) and Y306 (Cγ) over last 50 ns of simulation for individual CPE-CLDN4 dimers (CLDN4 subunits A, C, E, G, I, K; CPE subunits U, V, W, X, Y, Z) and average of all six. Bottom: SASA values (mean for last 50 ns) for the individual subunits (letters) and mean. Free: Mean value of L151 of the six claudins not interacting with this pocket in cCPE. (**F**) Cis-interface between two CLDN4 subunits. Top left: Cartoon of the interface, key residues are shown as sticks and interacting hydrophobic residues also as semi-transparent surface. CLDN4 subunits are colored cyan with ECS2 shown and blue with ECH shown, CPE is green. Top right: Distance between L71 (Cγ) and F147 (Cγ) over the last 50 ns of simulation for individual CLDN4 dimers (subunits given as letters) and average of all six. Bottom: SASA values (mean for last 50 ns) for the individual subunits (letters) and mean. Free: Mean value of corresponding residues in VIIIa-CPE_6_R_6_ simulation not consisting of CLDN4-CLDN4 interfaces.

The MD simulations of VIIIc-CPE_6_R_6_R´_6_ enabled a detailed analysis of all inter-subunit interactions that stabilize the complex (Fig. 10). The cap region of CPE was stabilized by two concentric β-barrels, where the inner one extends toward the transmembrane pore. Intramolecular contacts between the two β-sheets (β3, β5 and β2, β6, β7, respectively) were mainly maintained by hydrophobic interactions formed by V58, L63, F71, V118, T120, V128, Y130, V132, I161, and L163 (Fig. 10B).

In contrast, intermolecular interactions between CPEs were facilitated by H-bonds between adjacent β-strands and by electrostatic side chain interactions. Two CPE subunits were held together at the top between the inner and the outer β-barrel by the E67-K165 interaction (Fig. 10C). At the backside of the bottom of the outer β-barrel, an electrostatic network is formed by D48 and R137 of one CPE subunit and S60, Y129, and K131 of the neighboring subunit (Fig. 10D). D48 occupies a central position within this network, consistent with mutagenesis and biochemical data showing that D48 is essential for CPE prepore formation [79]. Thus, we proceeded with a quantitative analysis of the interface and measured the mean electrostatic interactions between D48 of one subunit and S60, Y129 and K131 of the neighboring subunit for all six interfaces within the last 50 ns of the simulation (Fig. 10D). The mean interaction counts (see methods Section 2.5) for the six CPE-CPE interfaces was ∼ 2.47 ± 0.25 interactions per ns, suggesting a significant contribution to the stabilization of the CPE oligomer.

The interaction between cCPE and CLDN4 was highly stable throughout the MD simulation, reproducing the hydrophobic and electrostatic interactions observed in the experimental cCPE/CLDN4 structures (PDB IDs 3X29, 5B2G, 6AKG, 6AKE, 6OV3, 6OV2, 7KP4, 8U4V; [35], [36], [37], [43], [61], [62]). In particular, L151 in the ECS2 of CLDN4 was tightly inserted into the triple tyrosine pocket of cCPE (Fig. 10E, [34], [35]). Distance measurement between L151 (Cγ, CLDN4) and Y306 (Cγ, CPE) over the last 50 ns of the simulation time revealed that the values remained nearly constant over time and between 5 and 6 Å with the exception of one interface, with a mean distance for all six CLDN4-cCPE interfaces of 5.85 ± 0.61 Å (Fig. 10E, top right). To quantify the hydrophobic interactions in this region, the solvent accessible surface area (SASA) of the involved residues (I258, A302, Y306, Y310, Y312 from CPE, and L151 from CLDN4) were calculated and normalized with respect to the number of C atoms in each residue. The exceptionally low values suggested significant water exclusion in the region across all interfaces (Fig. 10E, lower right). Notably, the SASA of L151 from CLDN4 which is embedded into the cCPE pocket, was 0.0 – 0.1, indicating total water exclusion. Similarly, the SASA of residue Y312 from CPE, which is present well inside the hydrophobic pocket, was nearly as low. Taken together, distance analysis and SASA values suggest that these residues form a highly hydrophobic interface that remains stable over time.

Finally, we analyzed claudin-claudin interactions within VIIIc-CPE_6_R_6_R´_6_. All claudin subunits remained attached to each other, although slight variations were evident during the simulation. Similar to the linear cis-interaction in claudin strands [52], [53], both electrostatic interactions and the proximity of hydrophobic residues were observed between ECS2 and ECH in two adjacent CLDN4 subunits, e.g., between E159 and S69 (Fig. 10F, top left). Thus, we termed this interaction “linear-cis-like interaction”. The distances between the Cγ atoms of L71 and F147 from two neighboring claudin subunits were calculated across all 12 interfaces. They remained stable among the interfaces over the last 50 ns of the simulation time, except for one interface which showed a slight distance increase from ∼ 6 to ∼ 8 Å (Fig. 10F, top right). Overall, the mean distance between L71 and F147 across all interfaces was ∼ 5.3 ± 1.0 Å, suggesting stable interaction similar to CLDN4 cis-interactions in claudin strands [52], [53]. This was further supported by the SASA values of residues involved in the linear-cis-like interactions, which were comparable to SASA calculations of linear cis-interfaces in the claudin strand models [52], [53]. For L71 in the ECH, which is the residue that fits into the hydrophobic pocket of the neighboring cis-interacting claudin, a low SASA value of 5.4 Å^2^ was calculated, whereas the neighboring L70 had a slightly higher mean SASA value of 6.6 Å^2^ (Fig. 10F, bottom). Further residues involved in hydrophobic interactions in the interface, namely F147 and M160, had slightly higher SASA than their counterparts in the linear cis-interaction in strand models. Of note, often cCPE residues also appeared to stabilize these claudin-claudin interfaces through interactions between cCPE-D213 and CLDN4-ECS2-K157, and cCPE-R208 with the CLDN4-ECS2 turn region (Fig. 10F, top left). Taken together, the distances and SASA indicate stable interactions in the linear-cis-like interfaces in the claudin ring of the CPE/claudin complex, which are partly comparable to linear cis-interactions in claudin strand models.

In summary, the MD simulations strongly supported the modeled architecture, claudin anchorage, membrane embedment, pore lining and cation selectivity for the CPE/claudin complex state VIIIc-CPE_6_R_6_R´_6_.

### 3.11 Site directed mutagenesis studies indicate interaction between claudin subunits during assembly of CPE pore complex

The VIIIc-CPE_6_R_6_R´_6_ model indicated an interaction between CLDN4 subunits similar to the highly conserved linear cis-interaction in polymeric claudin TJ strands [14], [52], [53], [80]. Previously, we showed that the glutamate mutation of S68 in CLDN3 (corresponding to S69 in CLDN4, Figure S10) at the conserved linear cis-interface inhibits claudin polymerization [59]. Given the high sequence similarity between CLDN3 and CLDN4 (89 %without the C-terminal cytoplasmic tail, Figure S10) and the fact that CPE binds strongly to both claudins [34], we took advantage of existing HEK293 cell lines expressing either mouse CLDN3 (mCLDN3) wild type or mCLDN3-S68E, but no endogenous CPE-binding claudins [34], [59], and established cellular binding and viability assays [15], [34]. We now tested whether weakening claudin cis-interaction through the S68E mutation affects the binding of cCPE to claudins and/or pore complex formation by CPE. Functional cytotoxic pore formation has a direct effect on cell viability, measured by the cells’ ability to metabolize MTT [81].

**Fig. 11:**
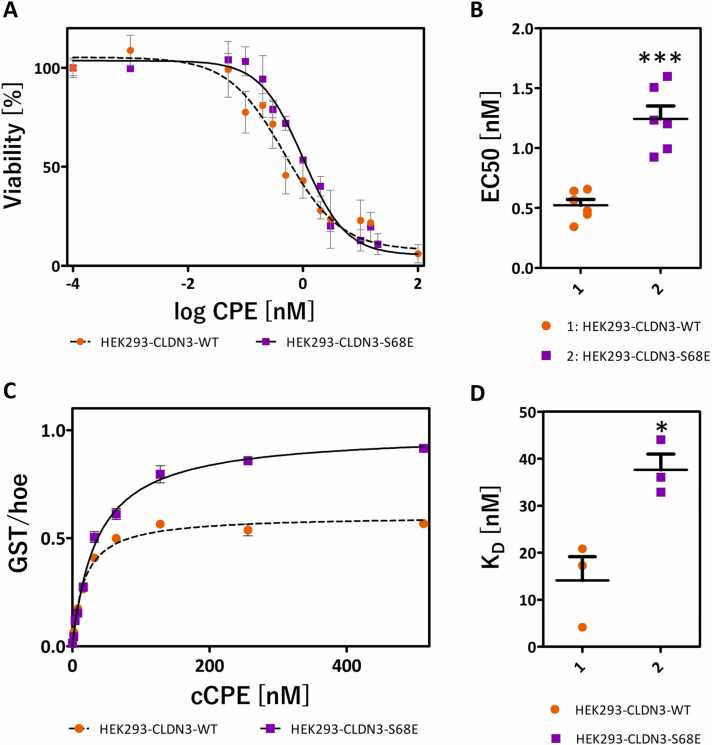
Cell viability and cCPE binding assays with HEK293 cells expressing either mouse CLDN3 wild type (mCLDN3-WT) or mCLDN3-S68E. **(A)** Viability of the HEK293 cells after 1 h incubation with different CPE concentrations tested by MTT assay. Normalized values (n = 3) were plotted against log of concentration (nM). **(B)** EC50 calculation from the data in (A) revealed a significantly higher EC50 value for HEK293 cells expressing the mCLDN3-S68E mutant. Mean ± SEM, n ≥ 6, *** p < 0.0003. **(C)** Cellular binding assay. HEK293-mCLDN3-WT, HEK293-mCLDN3-S68E cells, or non-transfected, claudin-free HEK293 cells were incubated for 30 min with different concentrations of GST-cCPE. After fixation, binding was detected by anti-GST antibodies and normalized to cell number using the Hoechst signal. From the binding data (n = 2), the unspecific binding (i.e., binding to non-transfected control cells) was subtracted. **(D)** Determination of K_D_ from the data in (C) and two replicates. The K_D_ was significantly higher for the mCLDN3-S68E mutant (38 ± 3 nM) than for WT (14 ± 5 nM). Mean ± SEM, n ≥ 3, * p < 0.05.

Both cell lines, HEK293-CLDN3WT and HEK293-CLDN3-S68E, were susceptible to a decrease in viability with increasing CPE concentration (Fig. 11A). However, the CLDN3-S68E mutation slightly prevented CPE-mediated cell damage, resulting in a higher EC_50_ value for cell viability (0.52 ± 0.05 nM for CLDN3WT and 1.24 ± 0.11 nM for CLDN3-S68E, Fig. 11B). Moreover, the CLDN3-S68E mutation slightly decreased the affinity of cCPE to the CLDN3-expressing HEK293 cells, as evident in binding saturation experiments (Fig. 11C). The dissociation constant (K_D_) of the mutant is 38 ± 3 nM, about twice the WT value of 14 ± 5 nM (Fig. 11D). In summary, the in vitro data show that both toxicity and CPE affinity are impaired when linear cis-interactions of claudins are inhibited by the S68E mutation and thereby support the idea that an interaction related to this linear cis-interaction contributes to the formation of claudin-anchored CPE-pore complexes.

## 4 Discussion

**Fig. 12:**
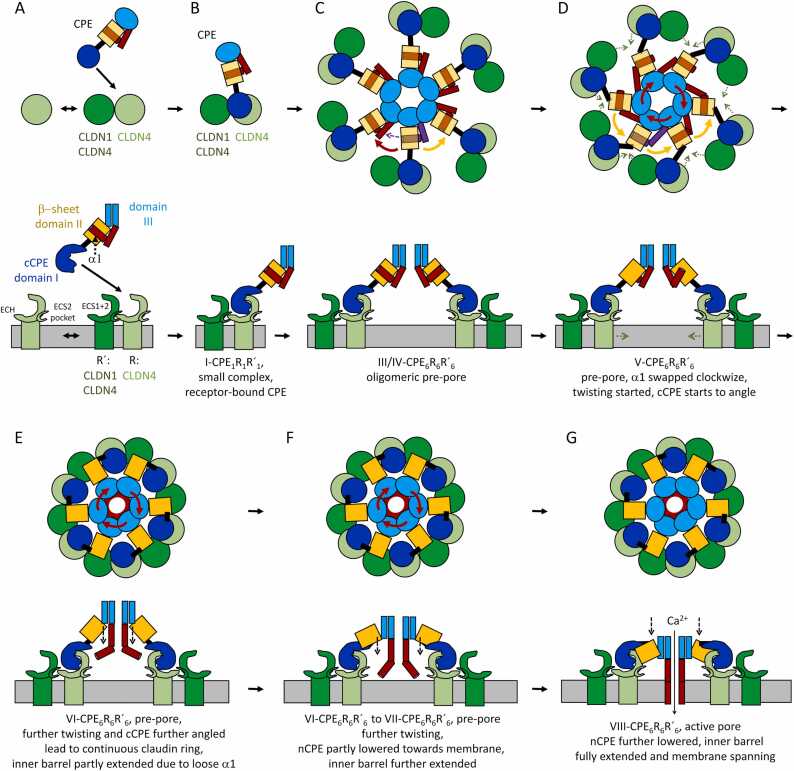
Schematic model of CPE/claudin pore complex assembly. Top views (top) and respective side views (bottom) of different stages. (**A**) Claudin dimers form dynamically. (**B**) Monomeric CPE binds to claudin dimer containing primary (CLDN4) and secondary receptor (e.g., CLDN1 or CLDN4). The trimeric complex (small complex) is stabilized by high affinity CLDN4-cCPE-, CLDN-CLDN cis-, and low affinity CLDN-cCPE interactions. (**C**) 6 small complexes oligomerize with minor conformational changes, only, into a large prepore complex containing 12 claudin and 6 CPE subunits. (**D**) α1-helix swaps clockwise (red arrows) from domain II in same CPE subunit to domain II in neighboring subunit. Concomitant anticlockwise movement (yellow arrows) of β-sheet in domain II, but not of the claudin receptors, support change of angle between domain II and cCPE. (**E, F**) α1-helix dissociates from the β-sheet in domain II leading to twisted extension of inner β-barrel. nCPE (domains II and III) starts to move downwards to membrane. cCPE angles further relative to nCPE. Claudins bound to cCPE move more to center leading to formation of a continuous claudin ring. (**G**) Further twisting movement of complex center against complex periphery drives full extension of the β-barrel, membrane penetration and formation of the active pore. For clarity, modeling intermediates (nCPE_6_ states, II-CPE_2–5_R_2–5_ and CPE_6_R_6_) are not depicted.

In this study, we predicted atomic models of different oligomerization states and derived mechanistic models for the prepore-to-pore transition of the cytotoxic CPE/claudin pore complex. Alignment of different structures predicted by the AlphaFold2 platform and experimentally solved CPE and cCPE/claudin structures were combined with published biochemical data on the CPE/claudin pore complex and MD simulations to drive model building. The key features of the developed model for the assembly of the CPE-pore-complex are schematically summarized in Fig. 12**:**

(1.) CPE monomers bind to claudin monomers or dimers containing one primary (e.g., CLDN4) and one secondary receptor (e.g., CLDN1 or CLDN4). A trimeric complex is stabilized by (a) the established high-affinity interaction between cCPE and the primary receptor claudin [34], [35], [36], (b) an experimentally indicated CLDN-CLDN interaction (modified linear cis-interaction, Fig. 10F), and a proposed additional low-affinity interaction between CPE and a secondary receptor claudin (Figs. 6B, 10F, 12B). This trimer is proposed to represent the small complex identified by co-immunoprecipitation analysis [40], [41], [75].

(2.) With only minor conformational changes, six of these small complexes oligomerize into a large prepore complex containing twelve claudin and six CPE subunits (Fig. 12C, states III-CPE_6_R_6_R′_6_ and IV-CPE_6_R_6_R′_6_). This stoichiometry was suggested by gel shift, size exclusion chromatography and co-immunoprecipitation analysis [41].

(3.) The α1-helix swaps clockwise from domain II in the same CPE subunit to domain II in the neighboring subunit. The concomitant anticlockwise movement of β-sheet in CPE domain II, but not of the claudin receptors, supports the change of angle between domain II and cCPE (Fig. 12D).

(4.) The α1-helix dissociates from the domain II β-sheet leading to a stepwise twisted extension of the inner β-barrel of CPE (state VI-CPE_6_R_6_R′_6_ to VIII-CPE_6_R_6_R´_6_, (Fig. 12E-G)).

(5.) Further conformational changes are required to form and drive the tips of the extending β-barrel through the membrane. nCPE has to get closer to the membrane without disturbing the cCPE-receptor (claudin) interaction. The model shows that this can be obtained by a conformational change of the flexible linker between cCPE (domain I) and nCPE (domains II and III) (Fig. 12E, F, G).

(6.) The diameter of the ring of twelve claudins has to be reduced to form the proposed continuous dodecameric claudin ring. The models show that this can be obtained by an additional conformational change in the cCPE-nCPE linker region resulting in a kink between cCPE and nCPE (Fig. 12D, E, F), and by a variant of the claudin-claudin cis-interaction (ECH-ECS2 pocket) that is suggested to drive polymerization of claudins within TJ strands [14], [52], [53]. We propose that the mechanistic advantage of such a receptor ring is that it may actively support a twist-like rotational movement of the central nCPE β-barrel against the interjacent CPE domain II and the peripheral cCPE-claudin ring of the complex. This might drive β-barrel extension and penetration through the membrane (Fig. 12D, E, F, G). Thus, our structural models provide a mechanistic model of the CPE-claudin pore formation to be further tested and validated by future studies.

The different components of the model are discussed below.

### 4.1 Formation of a hexameric CPE β-barrel pore

The model of the hexameric nCPE prepore state (state IV-nCPE_6_, Fig. 1D, E) shows a very high similarity to the HA3 subcomponent of the *C. botulinum* type C progenitor toxin hetero-oligomer (Fig. 3A, E). This state is highly reliable due to the highly similar fold of both proteins (RMSD for protein backbone of CPE_26–202_ and HA3a or HA3b: 1.79 Å), similar oligomeric arrangement (RMSD for protein backbone of IV-nCPE_6_ and HA3 oligomer: 3.14 Å) and formation of a similar concentric double-β-barrel as found in other aerolysin-like toxins [66], [67], [82]. In addition, the hexameric active pore states (VII-nCPE_6_, VIII-CPE_6_R_6,_ VIII-CPE_6_R_6_R´_6_) show a similar elongated β-barrel pore arrangement as other aerolysin-like toxins ([65], [66], [67], [82], Fig. 3). However, in contrast to aerolysin, the unique barrel consists of 12 β-strands originating from 6 CPE subunits instead of 14 β-strands originating form 7 toxins subunits. Importantly, the hexameric pore was stable during multiple MD simulations (for VII-nCPE_6_, VIIIa-CPE_6_R_6_, VIIIb-CPE_6_R_6_, VIIIc-CPE_6_R_6_R´_6_, and VIIId-CPE_6_R_6_R´_6_; Fig. 5, Fig. 8, S3, S5, S8). Compared to the corresponding heptameric CPE pore model, the hexameric one was more favorable (Figure S2) and was in much better agreement with the biochemical data that indicates a hexamer [41].

The reliability of the modeled pore β-barrel is furthermore supported by the following: (i) The overall mushroom-like architecture consisting of an elongated β-barrel stem (∼ 9 nm long and ∼ 3.5 nm wide) and a cap with a central concentric double β-barrel and receptor binding domains at the periphery is similar to other aerolysin-like toxins [27], [66], [67]. (ii) All membrane-facing residues are hydrophobic, and all pore lumen-lining residues are hydrophilic. (iii) Complete consistence to experimental data: (a) residues 80–106 form the membrane-spanning β-hairpin region with F91 and F95 at the tip (Figs. S3, Fig. 2, Fig. 5, Fig. 8), as shown by site-directed mutagenesis (region 80–106, residues F91, F95, G103 are critical for membrane insertion [42], [83]; (b) D48 participates directly in the CPE-CPE interface (Fig. 10D), fitting to its essential contribution to oligomerization [11]; (c) three unique hexa-glutamate rings along the ∼ 8 Å wide pore are formed by E101, E80 and E115, which strongly attract cations leading to charge selectivity. This agrees with the strong cation selectivity of the CPE pore demonstrated by electrophysiological measurements [69], [70].

In contrast to other members of the aerolysin family (like aerolysin, lysenin, or *C. perfringens* ɛ-toxin), the membrane-spanning region of CPE needs to undergo an α-to-β transition of secondary structure during pore formation. Thereby, the α-helix of nCPE domain II changes its conformation. Similar changes have been shown for β-barrel pore formation in cholesterol-dependent cytolysins, driven by π-stacking and electrostatic interactions, and have been described as a unique feature of the CDC/MACPF/SNTX (cholesterol-dependent cytolysin/membrane attack complex perforin/stonefish toxin) superfamily of PFTs [84]. The opposite, a β-to-α transition of the pore forming region, occurs in the α-PFT cytolysin A (ClyA) [85], [86].

Compared to the nCPE structures in the states IV-nCPE_6_, VII-nCPE_6_, VIIIa-CPE_6_R_6_ and VIIIc-CPE_6_R_6_R´_6_, the structures in the intermediate states V-nCPE_6_ and VI-nCPE_6_ (Fig. 1) are based on less corroborating data. However, by combining AlphaFold2 predictions with known structures, we were able to deduce a potential sequence of conformational changes in nCPE, from the monomeric CPE to the active transmembrane pore complex.

### 4.2 Comparison of AlphaFold2 and AlphaFold3 predictions

We also tested AlphaFold3 with the same CPE inputs as used for AlphaFold2 (sequences, number of subunits, see Table 1 in Section 2.2)**.** No meaningful hexamers or heptamers were obtained that contained a central prepore or pore formed by nCPE. Strikingly, addition of CLDN4 to CPE, either in a 1:1 or 2:1 stoichiometry, yielded prepore-like complexes with cCPE/CLDN4 interface very similar to the one in dimer crystal structures [36], [72]. However, in contrast to the AlphaFold2 models (AF-I to AF-V resulting in states IV-nCPE_6_ to VII-nCPE_6_), they did not contain a β-barrel formed by β3- and β5 strands of nCPE. Moreover, D48 was not part of an oligomeric interface and the resulting tilt of the claudins in the membrane was very different from the one in linear claudin strand models [52], [53], [78] (see Figure S11 for an example). Thus, the reliability for the AlphaFold3 prepore models is questionable. Not only in this context, a recent study focusing on the high-resolution structure of the dimeric CPE/CLDN4 complex revealed by cryo-EM is of particular interest [44]. Here, AlphaFold3 was used to generate CPE prepore models that are highly similar to our AlphaFold3 predictions. The authors propose that the tilted claudins in the AlphaFold3-predicted prepore complex could cause a positive membrane curvature that could support insertion of the membrane-spanning CPE segment during pore formation. Such membrane curvature could also facilitate that the β-hairpins in our pore model state VII-CPE_6_R_6_ are fully spanning the membrane (Fig. 4A) without our proposed linker re-arrangement.

### 4.3 CPE-claudin interaction

The anchorage of the CPE-hexamer to claudin receptors in the membrane fits very well with respect to topology relative to the membrane and to the known interaction sites between cCPE and claudins. In particular, the experimental biochemical and structural data identified key interactions (CLDN4-ECS2-P150/L151 with the Y306/Y310/Y312 pocket in cCPE, and CLDN4-ECS2-N149 with cCPE P311 backbone) that remained very stable throughout MD simulations of several membrane embedded oligomeric CPE/CLDN4 complexes (VIIIa-CPE_6_R_6_, VIIIb-CPE_6_R_6_, VIIId-CPE_6_R_6_R´_6_ and shown for VIIIc-CPE_6_R_6_R´_6_ in Fig. 10E). Thus, this CPE-claudin interaction is of high reliability. Of note, to our knowledge, this is the first time that stability of the interaction between CPE (26−319) and claudins was demonstrated on the atomic level under dynamic conditions. Interestingly, in the state VIIIc-CPE_6_R_6_R´_6_ containing the putative dodecameric CLDN4 ring, an additional interaction site was found between CPE and the secondary receptor claudin (cCPE-D213 with CLDN4-ECS2-K157 and cCPE-R208 with the CLDN4-ECS2 turn), leading to a trimeric CPE-CLDN4-CLDN4 interaction (Fig. 10A, E, F; 12B). Importantly, the secondary CPE-claudin interaction would not depend on the ECS2 interaction motif that is found only in primary high affinity CPE receptor claudins, such as CLDN3, −4, −6, −9, −14 [16], [39], [87]. Thus, CLDN1 could also be a secondary receptor, explaining why CLDN1 is robustly identified in CPE/claudin complexes despite its low affinity to cCPE [40], [41], [87], [88]. However, the validity of the proposed additional CPE-claudin interface as well as of the proposed claudin-claudin cis-interface for the small complex (I-CPE_1_R_1_R´_1_) and the active pore complex (VIIIc-CPE_6_R_6_R´_6_) remains to be tested. Also, other subunit arrangements are conceivable, for instance, with face-to-face claudin dimers [14] that would be hardly compatible with the formation of the proposed continuous claudin ring.

### 4.4 Conformational changes in the nCPE-cCPE linker region during CPE/claudin pore complex assembly

The nCPE pore predictions and simulations as well as the published cCPE-claudin structural interaction data appeared to be very reliable. However, combining both with the nCPE-cCPE linkage present in the crystal structure of monomeric CPE did result in a complex (VII-CPE_6_R_6_) in which the pore barrel does not fully span the membrane plane that is defined by the transmembrane domains of the claudins (Fig. 4A). Of note the β-strands β1, β2, β6, β7 that connect domain II and III in nCPE did not change strongly between the monomeric CPE and the pore states (VII-nCPE_6_, VII-CPE_6_R_6_, VIII-CPE_6_R_6_ and VIII-CPE_6_R_6_R´_6_, Fig. 1, Fig. 6, Fig. 7, S1). Thus, the most plausible conformational change to enable a transmembrane pore appeared to be a modification of the probably more flexible, unstructured nCPE-cCPE linker region. Consequently, in the first step, nCPE was moved mainly downwards relative to the cCPE/CLDN4 complex, which resulted in a complex with a stable and functional transmembrane pore (state VIIIa-CPE_6_R_6_, Fig. 4). In a second step, cCPE was further re-arranged relative to nCPE to fit the 6 CPE subunits to a continuous ring of 12 claudin subunits (state VIIIc-CPE_6_R_6_R´_6_, Fig. 6, Fig. 7, Fig. 8). Although these manual modifications extensively changed the overall CPE conformation, they did not alter the conformation of either the hexameric nCPE or the cCPE/CLDN4 complexes. In addition, in the CPE-related HA3 complex (Fig. 3E), the domain corresponding to cCPE is also angled slightly in the same direction. Due to the manual modification of the nCPE-cCPE linker region, details in the conformation of the linker and in the relative positioning of cCPE, nCPE and the secondary receptor claudin are more uncertain than other aspects of the complex. Nevertheless, as shown by MD simulations, the resulting structure (state VIIIc-CPE_6_R_6_R´_6_) led to stable and functional CPE/claudin pore complexes. Thus, the proposed conformational changes in the nCPE-cCPE linker provide a plausible option.

### 4.5 Driving force for transmembrane pore barrel formation

We propose that the formation of a receptor ring may actively support a twist-like rotational movement of the inner nCPE pore β-barrel against the outer cCPE-claudin ring. This could contribute to β-barrel extension and thereby its penetration through the membrane. The postulated overall CPE prepore-to-pore conversion resembles that of other aerolysin-like β-PFTs: Prepore formation, β-barrel expansion in a zipper-like manner and collapse by change of angle between the receptor-binding and the pore-forming domains [66], [89]. Interestingly, much like in our CPE model, conformational movements including rotation of central against peripheral domains and flattening of the complex have been observed for *C. perfringens* ε-toxin [90] and aerolysin [91]. In aerolysin, rotation of domain 4 potentially drives the circular association of β-sandwich domains of each monomer, leading to (prepore) β-barrel formation. This driving force hypothesis of aerolysin was derived through structural analysis of different states (prepore to final pore), analogous to our CPE study in which different states were modeled [27], [91]. In addition, alternating polar serine and threonine residues in the insertion loop of all β-PFTs may contribute to driving the prepore-to-pore transition by aiding membrane binding, oligomerization, and facilitating amphipathic loop insertion during transmembrane pore formation [66]. Further studies could take up a more in-depth, comparative analysis regarding this transition using our CPE model, which unlike other studies on PFTs, also strongly considers the toxin-receptor complex.

### 4.6 Diameter and selectivity of the CPE pore

When analyzing the mean pore diameter profile (Fig. 9D, E), we observed the minimal constriction zone adjacent to the E101 ring closes to the cytoplasmic side with a mean diameter of 7.8 Å (Fig. 9E). Benz and Popoff estimated that the diameter of a CPE channel could roughly be 14 Å, wider than in our model [70]. They determined this value through a fit of the single channel conductance as a function of the bulk solution. However, such a discrepancy in experimentally calculated pore diameters with the actual diameters in the resolved structures was already seen in the case of other β-PFTs [92]. For example, different studies reported varying diameters from 7 Å to 30 Å for the aerolysin pore (30 Å [93], 7 Å [94]; 9.3 Å [95]; ∼ 17 Å [96]; 17 Å [97]; 19–23 Å [98]), whereas the actual diameter observed in the aerolysin pore crystal structure (PDB ID: 5JZT) was 13.7 Å [66]. In addition, Benz and Popoff showed that the CPE pores are highly cation selective, whereas most other β-PFTs are either anion-selective or non-selective [70]. This unique cation selectivity is clearly evident through our model. Notably, CPE-induced cytotoxicity relies on increased influx of Ca^2+^ through the assembled CPE pore. Modest Ca^2+^ influx at relatively low CPE concentrations (∼ 1 μg/ml, in vitro) results in limited calpain activation and causes the classical caspase-3-dependent apoptosis [22], [99], while a massive calcium influx caused by higher pathophysiologic CPE concentrations triggers strong calpain activation, which has been shown to cause necroptosis, a form of necrosis [25], [100].

## 5 Conclusion

In summary, we predicted a model of CPE pore formation that is fully consistent with published structural, biochemical, and functional data. Limitations of this study are that it does not contain detailed experimental validation by in vitro structure-function analysis of CPE and claudin mutants nor a new experimentally solved complex structure. Very recently the structure of the dimeric CPE/CLDN4 complex was resolved by cryo-EM [44]. However, no larger (pre-)pore-like complexes were shown in this work. This underlines the experimental challenge of purifying and resolving CPE/claudin pore complexes. Thus, it is reasonable and constructive to use a computational prediction and simulation approach to elucidate structural and mechanistic aspects of the pore formation and charge selectivity of the CPE/claudin pore complex. This novel knowledge opens the way for further investigations on the structure, physiology, and pathophysiology of CPE and claudins. The structural insights gained could also be used to develop therapeutic agents to alleviate the clinical symptoms of severe cases of gastrointestinal disease caused by CPE. For example, virtual screening for pore blockers and further testing of identified candidates in cellular cytotoxicity assays could be a promising approach.

## CRediT authorship contribution statement

**Jörg Piontek:** Writing – review & editing, Writing – original draft, Visualization, Supervision, Project administration, Methodology, Investigation, Funding acquisition, Conceptualization. **Joy Weber:** Writing – review & editing, Validation, Methodology, Investigation, Formal analysis, Data curation. **Santhosh Kumar Nagarajan:** Writing – review & editing, Visualization, Validation, Methodology, Investigation, Formal analysis, Data curation. **Daniel Roderer:** Writing – review & editing, Supervision, Resources, Project administration, Methodology, Funding acquisition, Conceptualization.

## Declaration of Generative AI and AI-assisted technologies in the writing process

During the preparation of this work the authors used https://www.deepl.com/de/write in order to improve the language and the readability of the manuscript. After using this tool, the authors reviewed and edited the content as needed and take full responsibility for the content of the publication.

## Declaration of Competing Interest

The authors declare no conflict of interest.

## Appendix A Supplementary material

.

Movie S1 Conformational changes of nCPE hexamer model. This movie shows a morph from the prepore state IV-nCPE_6_ to prepore state V-nCPE_6_.

.

Movie S2 Conformational changes of nCPE hexamer model. This movie shows a morph from the prepore state V-nCPE_6_ to pore state VII-nCPE_6_.

.

Movie S3 Conformational changes of nCPE hexamer model. This movie shows a morph from the prepore state IV-nCPE_6_ to pore state VII-nCPE_6_.

.

Movie S4 MD simulation for pore state VII-nCPE_6_. The last 50 ns of simulation are shown.

.

Movie S5 MD simulation for pore state VIIIa-CPE_6_R_6_. The last 50 ns of simulation are shown.

.

Movie S6 MD simulation for pore state VIIIc-CPE_6_R_6_R´_6_. The last 50 ns of simulation are shown.
