# Supplementary material for: C. perfringens enterotoxin-claudin pore complex: Models for structure, mechanism of pore assembly and cation permeability
Source: Comput Struct Biotechnol J. 2024 Dec 2;27:287–306. doi: 10.1016/j.csbj.2024.11.048 (PMC11774686; doi:10.1016/j.csbj.2024.11.048)
Supplement: Supplementary file 1 — Supplementary material [file mmc1.pdf]

Supplementary information

C. perfringens enterotoxin-claudin pore complex: Models for structure, mechanism of pore assembly and cation permeability

Santhosh Kumar Nagarajan[1], *Joy Weber*[1, 2], *Daniel Roderer*[2,]*, *Jörg Piontek*[1,]*

[1] Clinical Physiology/Nutritional Medicine, Department of Gastroenterology, Rheumatology and Infectious Diseases, Charité – Universitätsmedizin Berlin, corporate member of Freie Universität Berlin and Humboldt-Universität zu Berlin, 12203 Berlin, Germany

[2] Leibniz-Forschungsinstitut für Molekulare Pharmakologie (FMP), Berlin, Germany

* Correspondence: joerg.piontek@charite.de, +49 30 450-514535; roderer@fmp-berlin.de, +49 30 94793 240

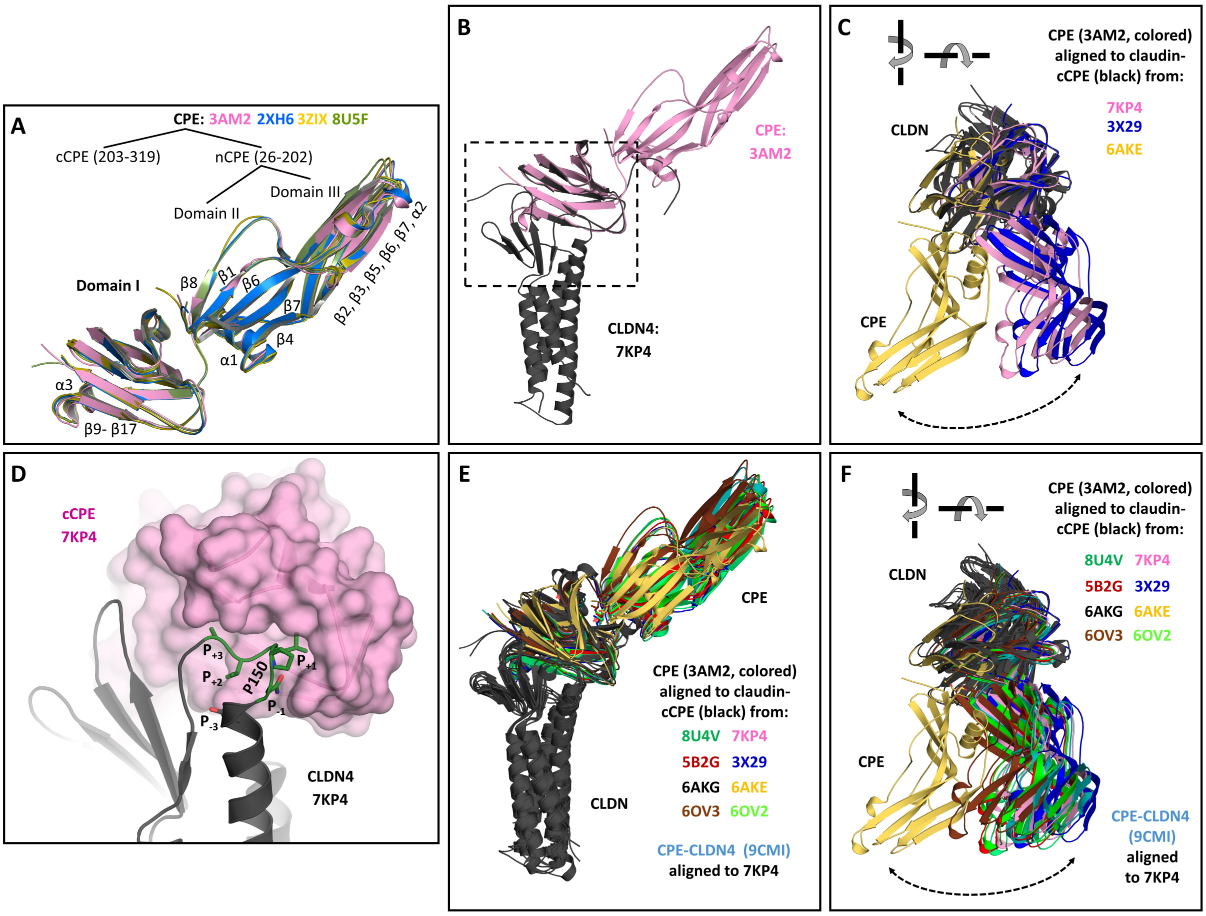

Figure S1 (A) Structural alignment of different CPE crystal structures. All structures show monomers and are highly similar. The nomenclature of secondary structure elements and domains follows Kitadokoro et al. [1]. For further analysis, PDB ID 3AM2 was selected as the representative structure. **(B-F)** Structural alignment of CPE (PDB ID 3AM2) to different cCPE-claudin complex structures. Each claudin chain was aligned to the claudin-4 chain from PDB ID 7KP4. Afterwards, the cCPE domain of a CPE chain was aligned to the respective cCPE chain of the cCPE-claudin complex. In addition, the CPE-CLDN4 complex (PDB ID 9CMI [2]) was aligned to the cCPE-CLDN4 complex (PDB ID 7KP4, **E,F**). Compared to B/E the view in C/F is rotated by ~90° in two directions (arrows). **(B, E)** The nine aligned CPE-claudin complexes do not differ concerning the angle of CPE towards the membrane plane (approximately orthogonal to the axis of the transmembrane helix bundle of the claudin). **(C, F)** In contrast, the complexes differ with respect to the rotational angle around the longitudinal axis of the transmembrane helix bundle of the claudin. **(C)** The cCPE-claudin complex structure used for the pore assembly model (PDB ID 7KP4) is shown in comparison to the two most extreme rotational variants (PDB IDs 6AKE and 3X29). The comparison of the different CPE-claudin complexes suggests that the binding of the cCPE domain to claudins allows a certain degree of rotational flexibility even for CPE as a rigid body. **(D)** A close-up of dashed box in (B) with 7KP4 in slightly different perspective, highlighting the interaction between CLDN4-ECS2 and cCPE. P150, which is conserved in classic claudins [3], is used as a reference position. The positions P_{-3}, P_{-1}, P, P_{+1}, P_{+2}, P_{+3} of the interaction motif (D/E($_{P-4}$) x($_{P-3}$) x($_{P-2}$) N($_{P-1}$)P($_{P}$) L/M/V($_{P+1}$) V($_{P+2}$) P/A($_{P+3}$)) are indicated and the corresponding residues shown as sticks. The key region P_{-1}, to P_{+3} is shown in green. cCPE is shown as a semi-transparent surface.

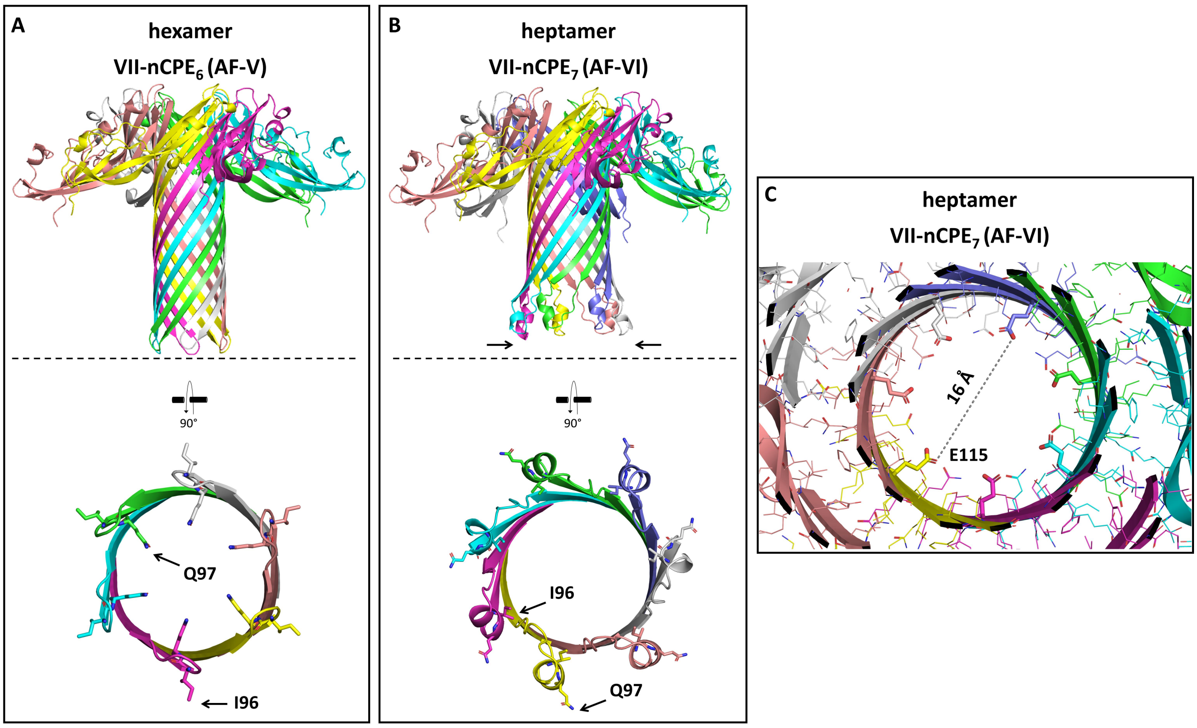

Figure S2: Comparison of AlphaFold2/ColabFold predictions (AF-V and AF-VI) for hexameric and heptameric variants of nCPE state VII. (**A, B**) Top: Side views of hexamer and heptamer. Note the inconsistent membrane-spanning region at the tip of the heptameric pore and its shorter length (arrows). Bottom: Top views on cross-sections through the tips of the transmembrane barrel of the hexamer and heptamer, viewed from the cytoplasmic side. I96 faces towards the hydrophobic membrane-embedded outside of the barrel in the hexamer but to the hydrophilic lumen in the heptamer. The opposite is observed for Q97. Lining, diameter and length of pore fits better to existing data for the hexamer. Both predictions (AF-V and AF-VI) are for $CPE_{26\text{-}202}$. AF-V shows the same architecture as AF-IV that was predicted for $CPE_{1\text{-}202}$. (**C**) Constriction at E115 ring in the cap region of the β-barrel of the heptamer model (16 Å). E115 residues are shown as sticks, others as lines.

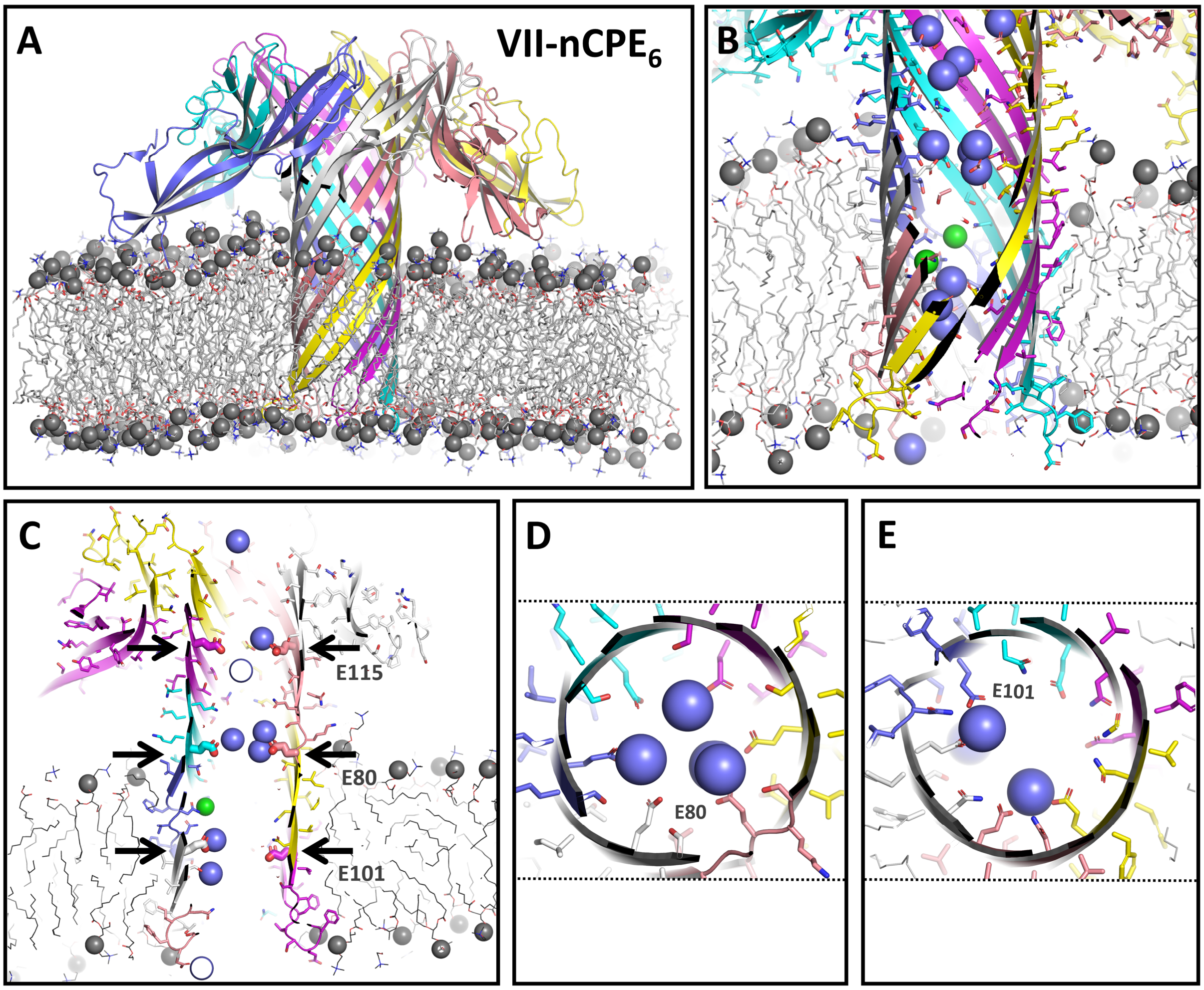

Figure S3. MD simulation of the hexameric nCPE pore. The protein complex state VII-nCPE$_6$ was embedded in a membrane (1-palmitoyl-2-oleoyl-sn-glycero-3-phosphocholine (POPC) lipid bilayer), equilibrated and simulated using Schrödinger Desmond. Snapshots after 100 ns of free simulation are shown with protein as cartoon, relevant side chains as sticks, lipid acyl chains as gray lines, phosphate head groups as gray spheres, sodium ions as blue and chloride ions as green spheres, respectively. (**A**) The pore barrel outside and inside the membrane and the pore cap are well preserved. The pore is well embedded in the membrane. (**B, C**) The pore is clipped in two versions to illustrate membrane embedment and pronounced presence of sodium ions in the pore. Rings formed by six E80 residues slightly above membrane plane (**D**) and by six E101 residues within the membrane plane (**E**) and by six E115 residues in cap region (see Figure S8E) strongly attract cations.

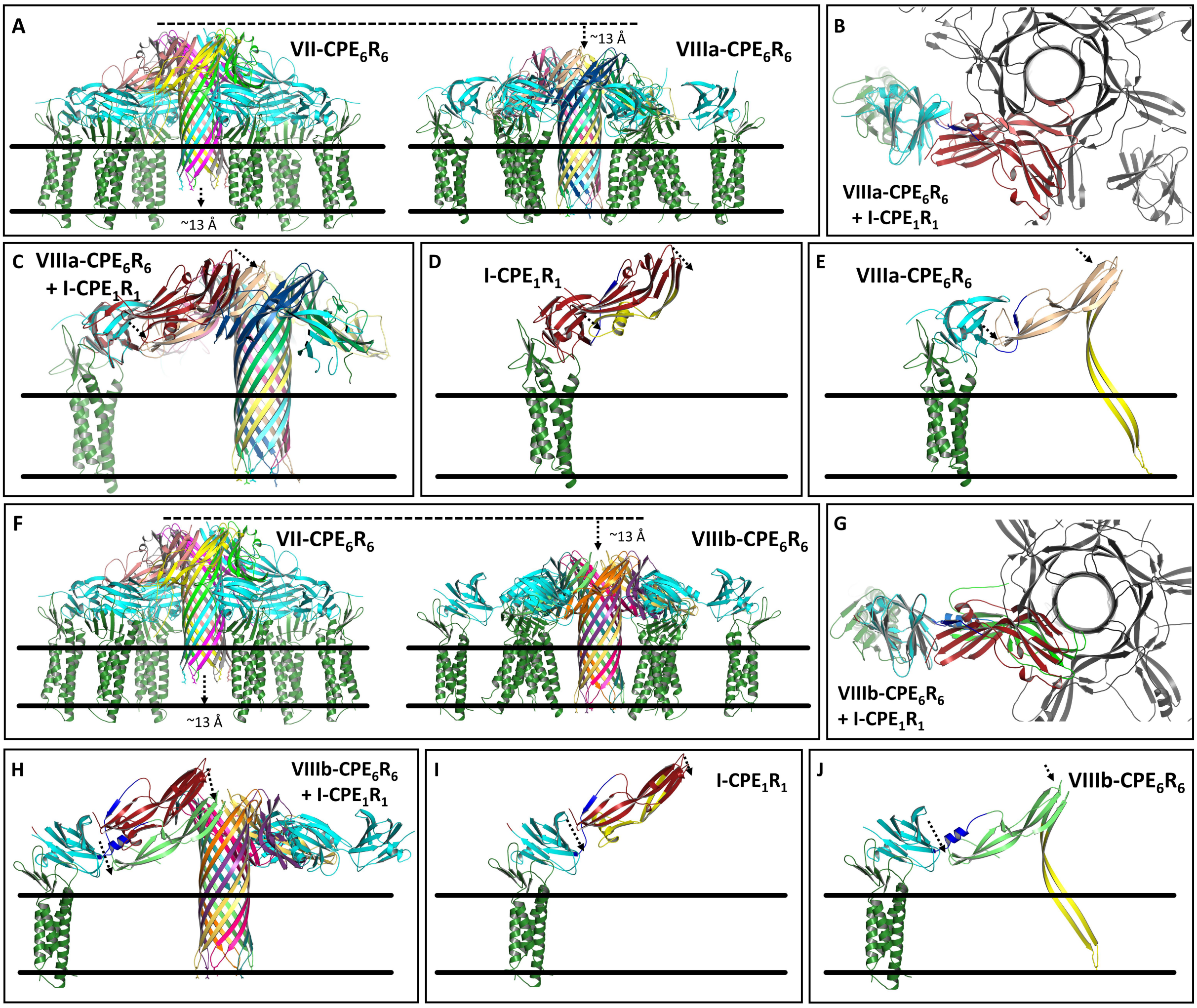

Figure S4: Conformational change in linker region between cCPE and nCPE (residues 191-205, blue) allowing full transmembrane penetration of the β-hairpin tips of the CPE pore in the context of claudin-bound CPE. Comparison of the two different conformational variants VIIIa-CPE_6R_6 (**A-E**) and VIIIb-CPE_6R_6 (**F-J**). (**A**) Comparison of CLDN4-bound CPE hexamer pore complexes without (state VII-CPE_6R_6) and with a downward shift of nCPE (state VIIIa-CPE_6R_6). E94 at the β-hairpin tip is shown as stick. (**B**) Top view of VIIIa-CPE_6R_6 with superimposed state I-CPE_1R_1 to visualize the conformational difference between the monomeric CPE structure (3AM2 of I-CPE_1R_1, red) and CPE in VIIIa-CPE_6R_6 (for one CPE subunit, nCPE is shown in pink and cCPE in cyan; the other five CPE subunits are depicted in gray). (**C**) Clipped view of state VIIIa-CPE_6R_6 focused on one claudin with superimposed state I-CPE_1R_1 to visualize the conformational difference between the monomeric CPE structure (3AM2 of I-CPE_1R_1, red) and CPE in VIIIa-CPE_6R_6 (cyan, beige). Individual CPE/claudin dimers are shown separately for I-CPE_6R_6 (**D**) and VIIIa-CPE_6R_6 (**E**). The region 73-116 that changes conformation is depicted in yellow in (D) and (E). In I-CPE_1R_1 it includes the α1-helix, in VIIIa-CPE_6R_6 it forms the main part of the pore β-barrel. The shift of nCPE in VIIIa-CPE_6R_6 relative to the position in I-CPE_1R_1 is labeled by dashed arrows in (C-E). **(F)** Comparison of CLDN4-bound CPE hexamer pore complexes without (VII-CPE_6R_6) and with variant downwards shift of nCPE (VIIIb-CPE_6R_6). (**G**) Top view on VIIIb-CPE_6R_6 with superimposed state I-CPE_1R_1 to visualize the conformational difference between the monomeric CPE structure (3AM2 of I-CPE_1R_1, red) and CPE in VIIIb-CPE_6R_6 (cyan, green for one CPE subunit, other CPE subunits in gray) (**H**) Clipped view of state VIIIb-CPE_6R_6 focused on one claudin with superimposed state I-CPE_1R_1 to visualize the conformational difference between the monomeric CPE structure (3AM2 of I-CPE_1R_1, red) and CPE in VIIIb-CPE_6R_6 (cyan, green). Individual

CPE/claudin dimers are shown separately for I-CPE_1R_1 (**I**) and VIIIb-CPE_6R_6 (**J**). The shift of nCPE in VIIIb-CPE_6R_6 relative to the position in I-CPE_1R_1 is labeled by dashed arrows in (H-J). Note that in VIIIa-CPE_6R_6 part of the nCPE-cCPE linker region (blue) still forms a β-strand (E) similar as in 3AM2 of I-CPE_1R_1 (D, I) whereas the corresponding linker region in VIIIb-CPE_6R_6 forms an α-helix above the β-sheet (β1, β6, β 7) in domain II (J).

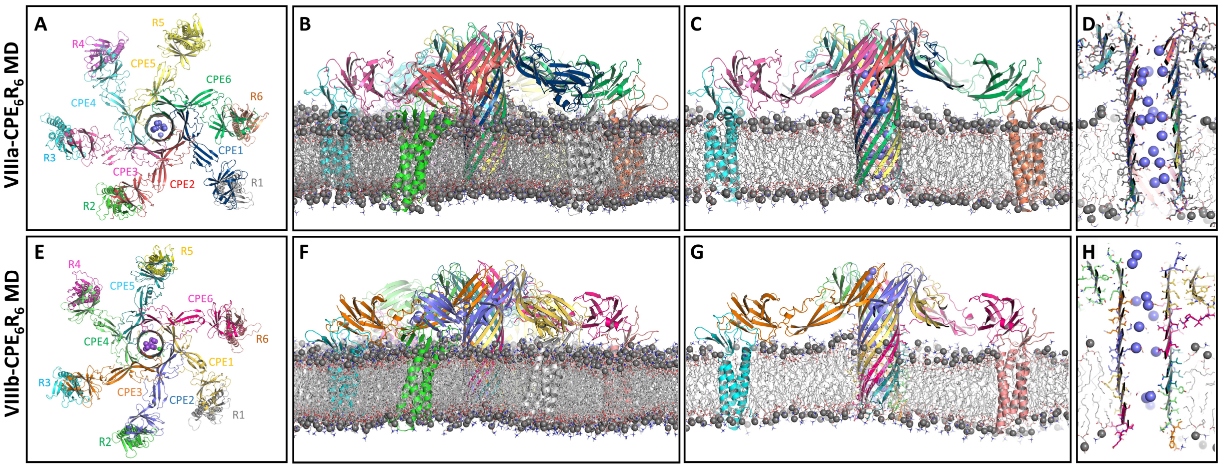

Figure S5: Comparison of MD simulations of CPE-CLDN4 pore complex states VIIIa-CPE_6R_6 (**A-D,** similar images as in Figure 4) and VIIIb-CPE_6R_6 (**E-H**). In both simulations, the pore β-barrel both outside and inside the membrane and the pore lining are well preserved. The pore β-barrel and the claudins remain well embedded in the membrane throughout the MD simulation, and the cCPE-claudin interaction is stable. See also legend of Figure 4.

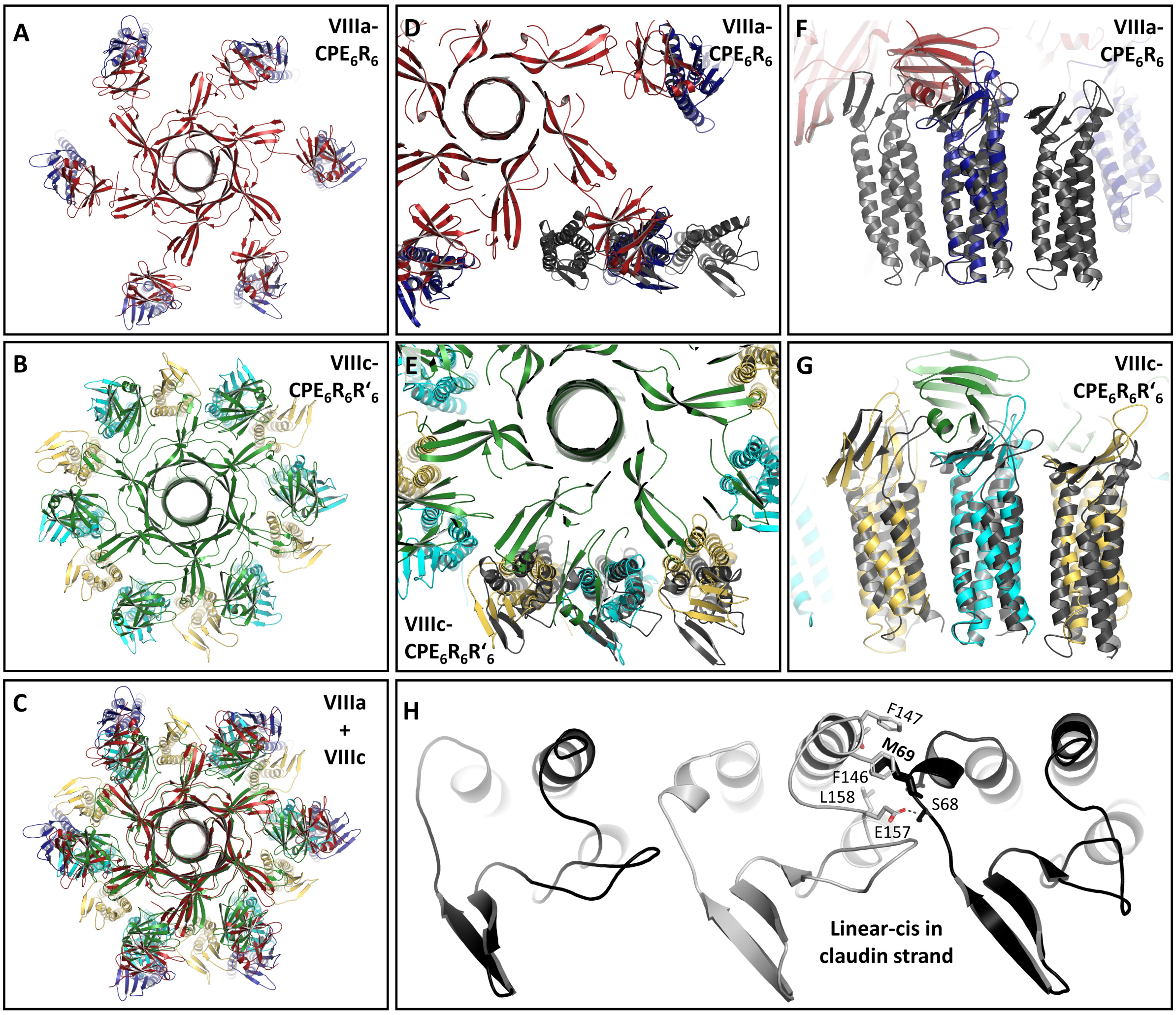

Figure S6: Manual addition of six more claudin subunits to generate a dodecameric claudin ring. (**A**) Top view of starting state VIIIa-CPE_6R_6 with six CPE (red) and six claudin (blue) subunits. (**B**) Top view of resulting state VIIIc-$CPE_6R_6R'_6$ with 6 CPE (green) and 12 claudin (cyan & yellow) subunits. (**C**) Top view of VIIIa-CPE_6R_6 and VIIIc-$CPE_6R_6R'_6$ superposition. The VIIIc-$CPE_6R_6R'_6$ model was generated according to the following hypotheses: (1) 12 claudins are part of the pore complex (biochemical evidence), and (2) the interface between the claudins could be a variation of the linear cis-interface found in claudin strands. In (**H**) the linear cis-interface of a CLDN10b strand model [4] is shown. Conserved key interfacial residues F146 (corresponding to CLDN4-F147), F147 (corresponding to CLDN4-Y148), L158 (corresponding to CLDN4-M160), E157 (corresponding to CLDN4-E159), S68 (corresponding to CLDN4-S69) and M69 (corresponding to CLDN4-L71) are shown as sticks. Conformational differences at cis-interfacial regions are caused by cCPE-binding (crystal structure evidence [3, 5, 6]). In the model state VIIIa-CPE_6R_6, the distance between CPE-bound claudins is too large to fit in a cis-interacting claudin (**D**), top view and (**F**), zoomed side view of VIIIa-CPE_6R_6 with superimposed linear-cis claudin trimer (gray). A planar CLDN4 dodecameric ring was manually docked based on 30° rotations of the linear cis-interface, shown as (**B**) top view, (**E**) zoomed top view, and (**G**) side view with superimposed linear-cis claudin trimer (gray). Six cCPE subunits were docked to each second CLDN4 subunit according to cCPE/CLDN4 structure (PDB ID 7KP4). nCPE was positioned close to the free CLDN4 subunits. This resulted in a shifted and turned position of the cCPE domain relative to the nCPE domain (see Figure 6). Both domains were connected by manually modifying the nCPE-cCPE linker region including the β8-strand).

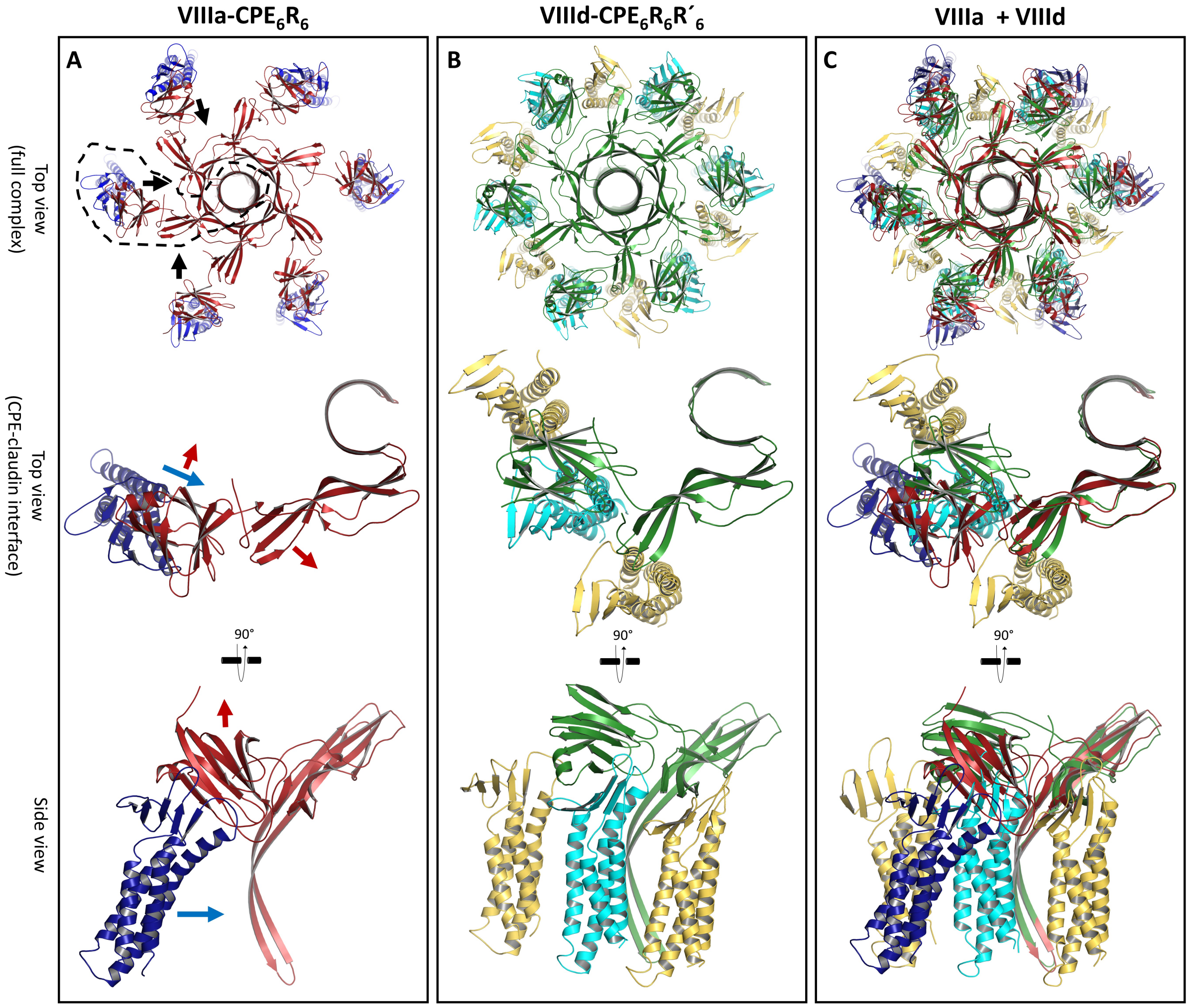

Figure S7: Details of modification of relative cCPE/nCPE positions for state VIIId-$CPE_6R_6R'_6$. (**A**) State VIIIa-CPE_6R_6: CPE pore hexamer (red) bound to six individual claudins (blue). (**B**) State VIIId-$CPE_6R_6R'_6$: CPE pore hexamer (green) bound to a dodecameric claudin ring. Claudin subunits primarily bound to CPE are colored cyan, and additional claudin subunits are colored yellow. (**C**) Superposition of VIIIa-CPE_6R_6 and VIIId-$CPE_6R_6R'_6$. Top views of complex (top), single CPE subunits of the complex bound to claudins are shown in top view (middle). For VIIIa-CPE_6R_6, one CPE subunit is bound to one claudin subunit. For VIIId-$CPE_6R_6R'_6$, both adjacent claudins are shown in addition to the primarily CPE-bound claudin subunit. The arrows indicate the direction of movement of cCPE (red), claudin (blue) and both (black) as they transition from state VIIIa-CPE_6R_6 to state VIIId-$CPE_6R_6R'_6$. Snapshots after 100 ns of MD simulation are shown.

VIIId-CPE$_6$R$_6$R´$_6$

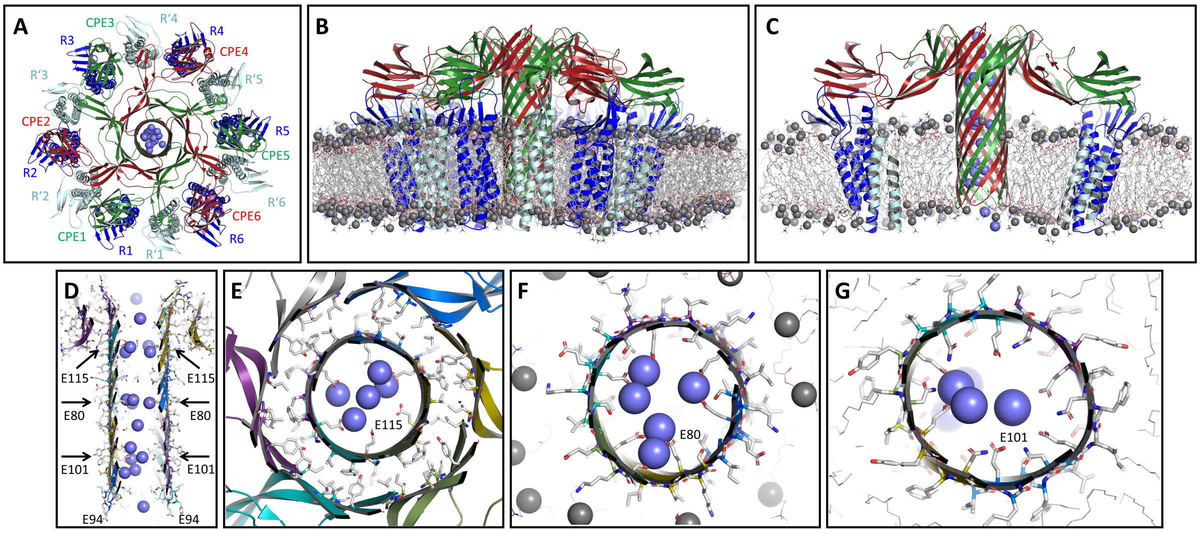

Figure S8: MD simulation of CPE hexamer anchored to dodecameric CLDN4 ring for pore complex state VIIId-CPE$_6$R$_6$R'$_6$, analogous as in Figure 8 for state VIIIc-CPE$_6$R$_6$R'$_6$. Snapshots after 100 ns of free simulation are shown with protein as cartoon, relevant residues as sticks, lipids acyl chains as gray lines and phosphate head groups as gray spheres, sodium ions as blue spheres. **(A)** Top view with CPE (CPE1-CPE6, alternating green and red), primary receptor CLDN4 (R1-R6, blue) and secondary receptor CLDN4 (R´1-R´6, cyan) subunits. (**B, C**) Side view (clipped in (C)). The pore barrel and the pore cap are well preserved. The pore complex is well embedded in the membrane. (**D**) Clipped side view of pore to illustrate pore lining and strong presence of sodium ions in the pore lumen. (**E**) The inner and outer β-barrels in the cap region (upper arrows in (D)) are held together mainly by interactions between hydrophobic residues. In the center, the ring of six E115 residues strongly attracts cations (Na^+ shown as spheres). Rings formed by six E80 residues (**F**) slightly above the membrane plane (middle arrows in (D)) and by six E101 residues (lower arrows in (D)) within the membrane plane (**G**) also strongly attract cations.

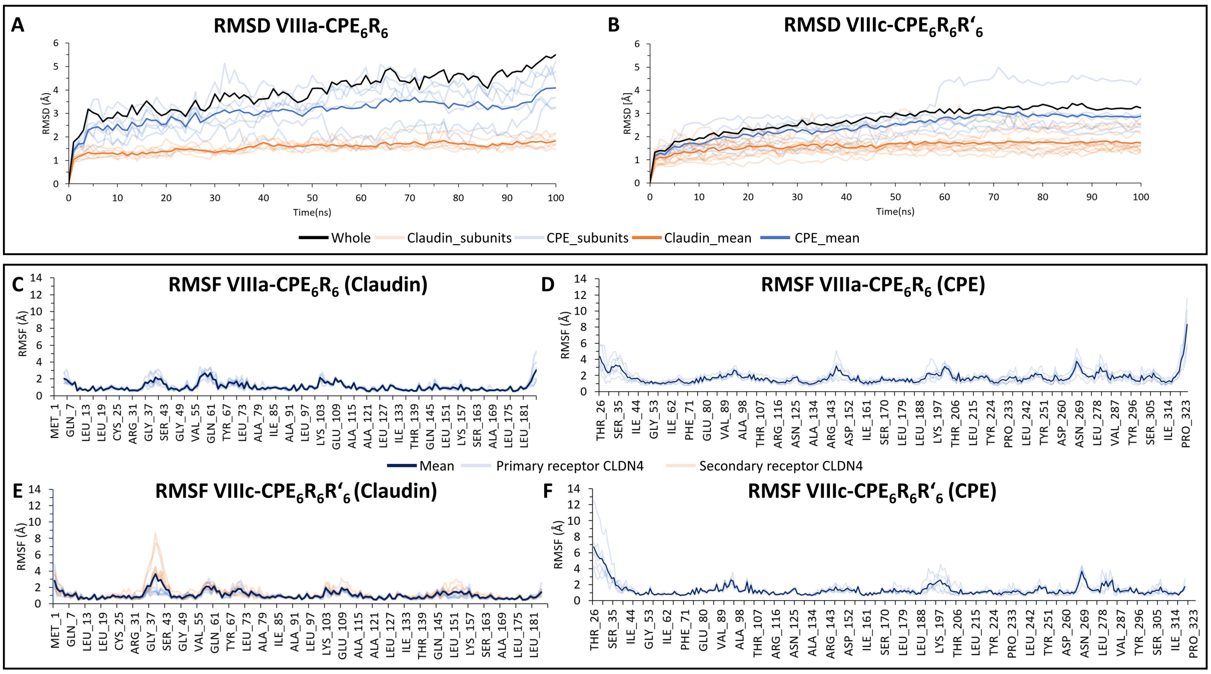

Figure S9: (A, B) Root-mean-square deviation (RMSD) of protein backbone for 6-claudin comprising VIIIa-CPE_6R_6 and 12-claudin comprising VIIIc-$CPE_6R_6R'_6$ pore complex states, respectively. The change in RMSD over the simulation time (100 ns) with respect to the starting structure (0 ns) is plotted. RMSD of the backbone of the whole VIIIa-CPE_6R_6 (including both CPE and claudins, shown in black) was varying slightly more than that of the VIIIc-$CPE_6R_6R'_6$. For VIIIa-CPE_6R_6, the discontinuous ring of six claudins resulted in higher flexibility, as evident because the RMSD varied until ~5.5 Å. On the contrary, for VIIIc-$CPE_6R_6R'_6$ with a closed 12-claudin ring, the RMSD reached saturation at ~3.0 Å after ~50 ns. Notably, the backbone of claudins (orange lines) in both complexes deviated around ~1.5 Å, which is comparable to the RMSD values of claudin backbones in previously published claudin strand models [4, 7]. Hence, the mean RMSD of the backbone of CPE subunits (blue lines), which fluctuated around ~3.0 Å and ~2.4 Å for VIIIa-CPE_6R_6 and VIIIc-$CPE_6R_6R'_6$, respectively, resulted in the variation in the whole RMSDs between both complexes. **(C** to **F)** Root mean square fluctuation (RMSF) of the amino acids of claudins and CPEs in both complexes. In both VIIIa-CPE_6R_6 and VIIIc-$CPE_6R_6R'_6$ (**C** and **E**), the fluctuations of residues in the claudins were mostly similar but differing mainly in the β1-β2 loop (residues 36-42) and ECS2 region (residues 146-156). To understand the difference, the RMSF values for primary receptor CLDN4 and secondary receptor CLDN4 subunits in VIIIc-$CPE_6R_6R'_6$ are given separately in addition (**E**). Here, we observed noticeable fluctuation in the residues of these regions for the secondary receptor CLDN4 subunits. Similarly, in the RMSF plots of the CPE subunits and their mean (**D** and **F**), fewer deviations were seen in certain regions in the CPE of VIIIc-$CPE_6R_6R'_6$ (residues 142 to 152), mainly due to the loose contacts of the CPE subunits with the additional secondary receptorCLDN4 subunits, which were not present in VIIIa-CPE_6R_6 due to the absence of claudins in the gaps. Expectedly, the manually-modified nCPE-cCPE linker segment (V187-A204) in VIIIc-$CPE_6R_6R'_6$ fluctuated slightly more than the average. A similarly noticeable higher fluctuation could be seen in the segment around N269, since it did not take part in any key intermolecular or intramolecular interaction. Overall, the mean RMSF for most residues in both claudins and CPEs was < 1.5 Å, underlining their stability within the complexes.

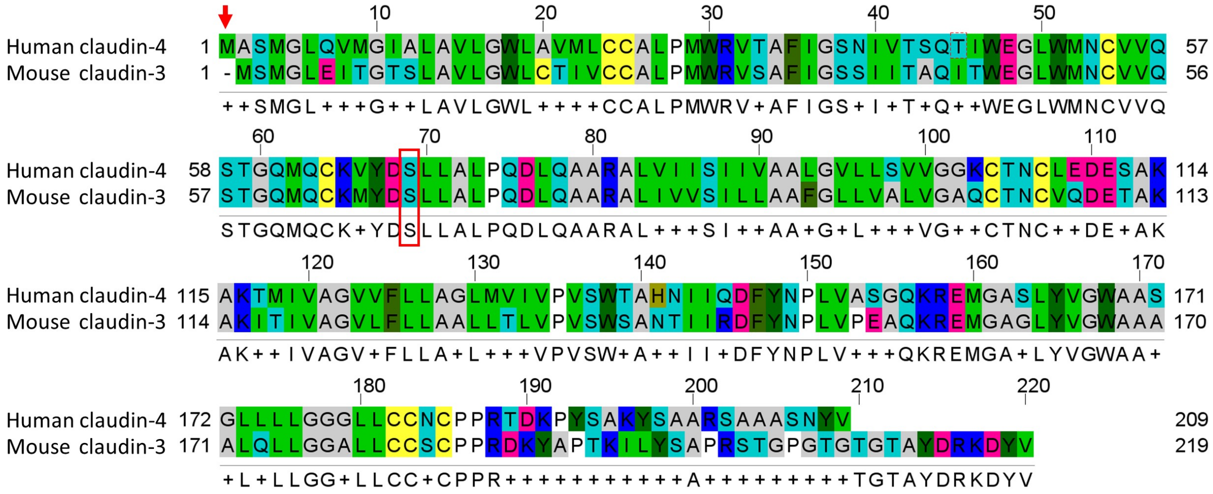

Figure S10: Sequence alignment of human claudin-4 and mouse claudin-3. Numbers (top) correspond to the positions in human claudin-4. Note that the mouse claudin-3 sequence is one amino acid residue shorter (red arrow at position 1). As a consequence, residue S68 in claudin-3 corresponds to residue S69 in claudin-4 (red box). The two sequences show high homology, in particular 72.3 % identity and 88.8% similarity in the region 1-188 (the highly variable C-terminal cytoplasmic region was excluded). The residues are colored according to their chemical properties. Below, non-identical residues are indicated by "+". The alignment was generated with Jalview version 2.11.4.1, Clustal WS [8].

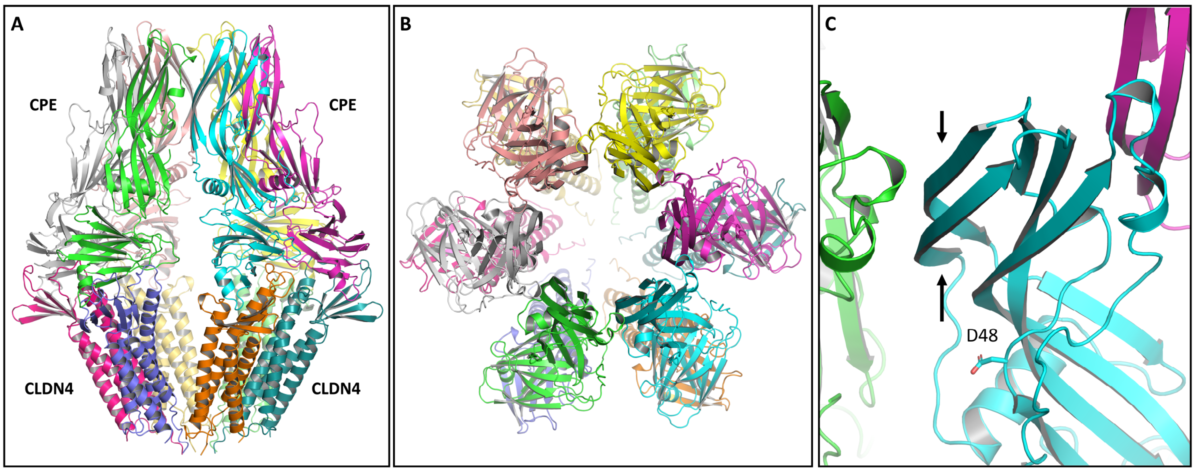

Figure S11: Example of an AlphaFold3 output. CPE and claudin subunits are shown as colored cartoons. **(A)** Side view. **(B)** Top view. **(C)** Close up of the interface between two CPE subunits. β3- and β5-strands (arrows) in nCPE are not forming a β-barrel, in contrast to the AlphaFold2-based CPE states reported in this study. D48 is not participating in an interface between neighboring CPE subunits. Six CPE-(26-319) and six hCldn4 (1-191) molecules were given as protein inputs using the AlphaFold3 Server (https://golgi.sandbox.google.com/).

Movie S1: Conformational changes of nCPE hexamer model. This movie shows a morph from the prepore state IV-nCPE$_6$ to prepore state V-nCPE$_6$. View from extracellular side. CPE subunits are shown as cartoon in different colors. See Figure 1 for more details.

Movie S2: Conformational changes of nCPE hexamer model. This movie shows a morph from the prepore state V-nCPE$_6$ to pore state VII-nCPE$_6$. View from extracellular side. CPE subunits are shown as cartoon in different colors. See Figure 1 for more details.

Movie S3: Conformational changes of nCPE hexamer model. This movie shows a morph from the prepore state IV-nCPE$_6$ to pore state VII-nCPE$_6$. View from extracellular side. CPE subunits are shown as cartoon in different colors. See Figure 1 for more details.

Movie S4: MD simulation for pore state VII-nCPE$_6$. The last 50 ns of simulation are shown. CPE subunits are shown as cartoon in different colors, Na^+ and Cl^- ions are shown as red and green spheres, respectively. Membrane lipids and water are not shown for clarity. Side view (large) and top view from extracellular side (small). The hexameric β-barrel pore is stable throughout the simulation and strongly attracts Na^+ ions.

Movie S5: MD simulation for pore state VIIIa-CPE$_6$R$_6$. The last 50 ns of simulation are shown. CPE and claudins subunits are shown as cartoon in different colors, Na^+ and Cl^- ions are shown as red and green spheres, respectively. Membrane lipids are shown as lines with phosphate atoms as pink spheres. Clipped side view (large) and full top view from extracellular side (small). The complex of six CPE and six CLDN4 subunits including the hexameric CPE β-barrel pore is stable throughout the simulation. The pore strongly attracts Na^+ ions.

Movie S6: MD simulation for pore state VIIIc-CPE$_6$R$_6$R´$_6$. The last 50 ns of simulation are shown. CPE and claudin subunits are shown as cartoon in different colors, Na^+ and Cl^- ions are shown as red and green spheres, respectively. Membrane lipids are shown as lines with phosphate atoms as pink spheres. Clipped side view (large) and full top view from extracellular side (small). The complex of six CPE and twelve CLDN4 subunits including the hexameric CPE β-barrel pore is stable throughout the simulation. The pore strongly attracts Na^+ ions.

Supplementary Methods

CPE protein preparation

CPE-Strep was expressed in *Escherichia coli* Rosetta-2. After expression was induced using 0.5 mM IPTG and subsequently 4 h of incubation at 37°C, CPE was purified from lysates using Strep-Tactin®XT columns (IBA, Göttingen, Germany). Bacteria from 1 l culture volume were harvested by centrifugation (12 min; 4500xg; 4°C) and resuspended in 50 ml lysis buffer (100 mM Tris/HCl; 150 mM NaCl; 1 mM EDTA, pH 8.0) additionally containing protease inhibitor cocktail (Merck). Lysis was performed with a LM10 Microfluidizer (Microfluidics, Westwood, USA) on ice. Cell debris was removed by centrifugation (30 min; 20000×g; 4°C) and supernatant loaded onto columns containing 2 mL Strep-Tactin® Superflow® high-capacity resin (IBA, Göttingen, Germany). The column was washed (with 10 ml lysis buffer) and CPE eluted with lysis buffer containing an additional 50 mM Biotin. The protein concentration was determined by NanoDrop Microvolume UV-Vis Spectrophotometers based on the absorbance at 280 nm.

GST-cCPE protein preparation

Plasmids carrying the open reading frame for GST-cCPEwt194−319 (GST-cCPE) have been previously documented [9]. Fusion proteins of GST-cCPE from these plasmids were produced in *E. coli* BL21 and purified according to established methods. The bacteria were cultivated until they reached an optical density of 0.6−0.8, at which protein expression was triggered by adding 1 mM isopropyl-β-D-thiogalactopyranoside. Three hours after induction, the bacterial cells were collected and lysed in lysis buffer (phosphate-buffered saline (PBS) with 1% (v/v) Triton X-100, 0.1 mM phenylmethylsulfonyl fluoride, 1 mM ethylenediaminetetraacetic acid, along with a protease inhibitor cocktail (Merck)). The cells were then sonicated with 15 pulses of 1 second each using a Vibra Cell Model 72434 BioBlock Scientific sonicator. The insoluble debris was separated by centrifugation at 20,000 × g for 30 minutes at 4°C. The GST-proteins were purified from the supernatant using glutathione agarose (Sigma-Aldrich) and subsequently dialyzed against PBS. The protein concentration was measured using the Pierce™ BCA Assay Kit (Thermo Fisher Scientific, Waltham, Massachusetts, USA).

Cell viability assays

To analyze CPE-mediated cell damage on HEK293 cells expressing the CLDN3-S68E mutant in comparison to HEK293-CLDN3WT cells, a viability assay was performed using 4,5-Dimethylthiazol-2-yl)-2,5-diphenyltetrazolium bromide (MTT). $5*10^4$ cells per well were seeded on 96-well plates coated with PLL and kept at 37°C, in 5% CO2. At about 95% confluence (after 24 h), the cells were incubated for 1 hour with variable dilutions of Step-

tagged CPE or 0.01% (v/v) Triton X-100 (negative control) before subsequently medium was replaced by medium without phenol red containing MTT (1.25 mM). After an additional 3-hour incubation, extraction and solubilization of water-insoluble blue-violet formazan was performed by treatment with 5% (v/v) Triton X-100 in 2-propanol for 20 minutes. Absorption was measured at 560 nm. The sample size was set to 6. For each concentration of each assay, three technical replicates (n=3) were prepared. The half-maximal effective concentration (EC50) values were calculated from the normalized data with the GraphPad Prism software using the model "log(agonist) vs. response" model (four parameter). A paired *t*-test was performed to determine significant differences between the EC50 values of Hek293-CLDN3-WT and Hek293-CLDN3-S68E.

Cellular binding assays

$3*10^5$ cells were seeded to PLL-coated 24-well plates and after 24 hours of cultivation at 37°C, in 5% CO2, the assay was performed at a confluence of about 90 to 100 %. Medium was exchanged with variable dilutions of GST-cCPE (0, 2, 4, 8, 16, 32, 64, 128, 256, 512 nM) in cell culture medium (0.5 ml per well). After a 30 min incubation at 37 °C and 5 % CO_2, GST-cCPE was removed and the cells were fixed (4% [w/v] paraformaldehyde, 10 min), followed by washing with PBS. For the subsequent quenching, 0.25 ml quenching buffer (0.1 M Glycin in PBS) was added to each well. Bound GST-cCPE was detected via PhycoLink® anti-GST-R-phycoerythrin conjugate; or alternatively in one case with anti-GST first antibody and Alexa594-linked 2nd antibody. The signal was normalized to cell number (Hoechst 33342). Each well was treated with 0.25 ml blocking reagent (1%(w/v) BSA, 0.05 %(v/v) Tween-20 in PBS) with PhycoLink® anti-GST-R-phycoerythrin conjugate (1:250) as well as Hoechst 33342 (2 µM) and incubated for 1h; or alternatively with 0.25 ml blocking reagent with the first antibody (1:250) and subsequently after 1h incubation, with 0.25 ml blocking reagent with the secondary antibody (1:500) and Hoechst 33342 (2 µM), followed by another 1h incubation. Normalized fluorescence intensity of bound anti-GST antibody was plotted against GST-cCPE concentration for CLDN3WT as well as the mutant, CLDN3-S68E.The K_D was calculated using nonlinear regression analysis for a single-site, specific binding in GraphPad Prism version 7.0 (San Diego, CA, USA). Unspecific binding was accounted for by subtracting the fluorescence signal after incubation of untransfected HEK293 cells with respective concentrations of GST-cCPE. Finally, a *t*-test was performed to determine significant differences between K_D values of GST-cCPE binding to Hek293-CLDN3WT or Hek293-CLDN3-S68E. For each assay two technical replicates of each well were prepared, and the assay was performed three times.

[1] Kitadokoro K, Nishimura K, Kamitani S, Fukui-Miyazaki A, Toshima H, Abe H, et al. Crystal structure of Clostridium perfringens enterotoxin displays features of {beta}-pore-forming toxins. JBiolChem. 2011;286:19549-55.
[2] Rathnayake SS, Erramilli SK, Kossiakoff AA, Vecchio AJ. Cryo-EM structures of Clostridium perfringens enterotoxin bound to its human receptor, claudin-4. Structure (London, England : 1993). 2024;32:1936-51 e5.
[3] Piontek J, Krug SM, Protze J, Krause G, Fromm M. Molecular architecture and assembly of the tight junction backbone. Biochimica et biophysica acta Biomembranes. 2020;1862:183279.
[4] Nagarajan SK, Klein S, Fadakar BS, Piontek J. Claudin-10b cation channels in tight junction strands: Octameric-interlocked pore barrels constitute paracellular channels with low water permeability. Comput Struct Biotechnol J. 2023;21:1711-27.
[5] Saitoh Y, Suzuki H, Tani K, Nishikawa K, Irie K, Ogura Y, et al. Tight junctions. Structural insight into tight junction disassembly by *Clostridium perfringens* enterotoxin. Science (New York, NY). 2015;347:775-8.
[6] Nakamura S, Irie K, Tanaka H, Nishikawa K, Suzuki H, Saitoh Y, et al. Morphologic determinant of tight junctions revealed by claudin-3 structures. Nat Commun. 2019;10:816.
[7] Nagarajan SK, Piontek J. Molecular Dynamics Simulations of Claudin-10a and -10b Ion Channels: With Similar Architecture, Different Pore Linings Determine the Opposite Charge Selectivity. Int J Mol Sci. 2024;25.
[8] Waterhouse AM, Procter JB, Martin DM, Clamp M, Barton GJ. Jalview Version 2--a multiple sequence alignment editor and analysis workbench. Bioinformatics. 2009;25:1189-91.
[9] Protze J, Eichner M, Piontek A, Dinter S, Rossa J, Blecharz KG, et al. Directed structural modification of Clostridium perfringens enterotoxin to enhance binding to claudin-5. Cellular and molecular life sciences : CMLS. 2015;72:1417-32.
